# Agomelatine, a Melatonin-Derived Drug, as a New Strategy for the Treatment of Colorectal Cancer

**DOI:** 10.3390/antiox12040926

**Published:** 2023-04-13

**Authors:** Sara Moreno-SanJuan, Jose D. Puentes-Pardo, Jorge Casado, Julia Escudero-Feliu, Huda Khaldy, Javier Arnedo, Ángel Carazo, Josefa León

**Affiliations:** 1 Cytometry and Microscopy Research Service, Biosanitary Research Institute of Granada (ibs.GRANADA), 18012 Granada, Spain; 2 Biosanitary Research Institute of Granada (ibs.GRANADA), 18012 Granada, Spain; 3 Department of Pharmacy, University of Granada, 18011 Granada, Spain; 4 Fundamental Biology Service, Scientific Instrument Center, University of Granada, 18071 Granada, Spain; 5 Department of Statistics and Operations Research, University of Granada, 18071 Granada, Spain; 6 Clinical Management Unit of Microbiology, San Cecilio University Hospital, 18006 Granada, Spain; 7 Clinical Management Unit of Digestive Disease, San Cecilio University Hospital, 18006 Granada, Spain

**Keywords:** agomelatine, melatonin, p53, circadian clock, colorectal cancer, SIRT1

## Abstract

The potential use of agomelatine as an alternative treatment for colorectal cancer is evaluated in this work. The effect of agomelatine was studied in an in vitro model using two cell lines with different p53 statuses (HCT-116, wild-type p53, and HCT-116 p53 null) and an in vivo xenograft model. The inhibitory effects of agomelatine and melatonin were stronger in the cells harboring the wild-type p53, although in both cell lines, the effect of agomelatine was greater than that of the melatonin. In vivo, only agomelatine was able to reduce the volumes of tumors generated by the HCT-116-p53-null cells. Both treatments induced changes in the rhythmicity of the circadian-clock genes in vitro, albeit with some differences. Agomelatine and melatonin regulated the rhythmicity of Per1-3, Cry1, Sirt1, and Prx1 in the HCT-116 cells. In these cells, agomelatine also regulated Bmal1 and Nr1d2, while melatonin changed the rhythmicity of Clock. In the HCT-116-p53-null cells, agomelatine regulated Per1-3, Cry1, Clock, Nr1d2, Sirt1, and Prx1; however, melatonin only induced changes in Clock, Bmal1, and Sirt1. The differences found in the regulation of the clock genes may explain the greater oncostatic effect of agomelatine in CRC.

## 1. Introduction

Colorectal cancer (CRC) is the third most common type of cancer and the second in terms of mortality worldwide [1]. The current treatments for CRC include 5-fluorouracil (5-FU) as a standard agent for chemotherapy, either as a single agent or in combination with other drugs, such as oxaliplatin or irinotecan [2]. However, these treatments have several limitations, such as severe side effects and the development of chemoresistance, which lead to unsatisfactory results [3]. These limitations have encouraged the development of the so-called targeted therapies, which are based on targeting concrete pathways according to each patient’s characteristics. However, the clinical application of these therapies is far from being established because they do not provide an improvement in cost–benefit terms, they still possess adverse effects, they differ in efficacy between patients with similar characteristics, and resistance has been shown to arise [2,3]. Therefore, there is a necessity to develop new approaches to improve or replace the current treatments.

Circadian rhythms are cyclic biological processes with a duration of close to 24 h, which are implicated in the regulation of major physiological events, such as the sleep –wake cycle, reproduction, and inflammatory and immune responses, among others. At the molecular level, the circadian rhythm consists of transcription–translation feedback loops (TTFL). The transcription-factor brain and muscle ARNT-like (BMAL-1) dimerizes circadian locomotor cycles kaput (CLOCK) and stimulates the transcription of Period 1-3 (Per1-3), Cryptocrome 1-2 (Cry1-2), the nuclear receptor subfamily 1 group D member 2 (Nr1d2), and RAR-related orphan receptor alpha (RORα) genes. In turn, the PER1-3 and CRY1-2 proteins form complexes in the cytoplasm and, when their concentration reaches a certain level, they translocate to the nucleus, where they inhibit the action of BMAL-1 and CLOCK and, thus, the transcription of their own genes. The PER/CRY complex is degraded, allowing the cycle to be restored every 24 h [4]. 

The circadian clock is not only based on transcriptional mechanisms; post-translational modifications of core circadian genes have also been described. Sirtuin 1 (SIRT1) is a nicotinamide adenine dinucleotide (NAD+)-dependent class III histone deacetylase, which is involved in cellular redox control [5]. It regulates the circadian clock at the central-nervous-system level by activating BMAL1 and CLOCK transcription [6]. At the peripheral level, it deacetylates BMAL1, affecting its activity [7], and PER2, preventing its dimerization with CRY [8]. It can also bind to CLOCK/BMAL1 complexes, which alters the expression of other associated genes, such as PER, in a manner that is probably related to the variable concentration of its co-enzyme, NAD+ [9]. In addition, the existence of circadian rhythms independent of the canonical molecular clock has been demonstrated. Specifically, there is a circadian rhythm of oxidation-reduction of peroxiredoxin-1 (PRX1), an antioxidant protein that scavenges hydrogen peroxide. This cycle is interconnected with the TTFL cycle, and both are individually necessary for the maintenance of rhythms at the cellular level [10]. In fact, it has been demonstrated that SIRT1 can activate PRX1 transcription [11]. The human PRX1 gene is a target of nuclear factor erythroid 2 related factor-2 (Nrf2) [12], a transcription factor shared by several antioxidant enzymes [13]. Furthermore, SIRT1 increases Nrf2 activation and decreases its polyubiquitination by decreasing the expression of Kelch-like ECH-associated protein 1 (Keap1)/Cullin 3 (Cul3) and by increasing Nrf2’s binding ability to anti-oxidant response element (ARE) [14]. 

Alterations in circadian rhythmicity have been linked to the onset and development of cancer [15]. Furthermore, the tolerability and efficacy of radiotherapy and chemotherapy also depend on circadian rhythms [16]. These experimental findings have raised interest in manipulating rhythms to prevent malignant transformation, to develop novel treatment strategies, and ultimately improve the outcomes of cancer patients [17]. In this sense, melatonin is an indolic compound secreted mainly by the pineal gland, although it can also be found in extrapineal tissues; it plays a key role in the control of circadian rhythms [18]. Melatonin exerts oncotastic properties in several cancer types. Regarding CRC, melatonin reduces tumour growth and proliferation and induced apoptosis in in vitro [19,20] and in vivo models [21]. Some of melatonin’s functions are mediated by the melatonin receptors MT1 and MT2. In fact, in CRC patients showed decreased levels of MT1 and MT2, without changes in melatonin levels, compared to their paired mucosa, in association with gender and invasion [22]. In addition, MT1 and MT2 levels are negatively correlated with cancer-stem-cell markers (CSCs), considered as the origins and major contributors to cancer progression, metastasis, and therapy resistance [23]. Taken together, these reports suggest that the use of non-selective MT1/MT2 agonists may be an interesting new approach for the treatment of CRC.

Agomelatine is a naphthalene analogue of melatonin, which shows agonist activities toward MT1/MT2 receptors, as well as greater affinity with them than melatonin, and antagonist activity toward the serotonin 5-HT2c receptor, and its use as an antidepressant drug has been approved [24]. Different in vivo models have proven that agomelatine synchronizes circadian rhythms, probably through MT1/MT2-receptor interaction [25]. The activation of MT1/MT2 synergically works with the blockage of the 5-HT2c receptor, resulting in neurogenesis induction, synaptic remodeling, and glutamate signaling, which explains agomelatine’s antidepressant role [24]. Curiously, serotonin has been associated with the progression of several cancer types, including CRC, and serotonin-receptor inhibitors may be used as therapy [26]. Along with the aforementioned influence of circadian rhythms and MT1/MT2 on cancer development and progression, this suggests that agomelatine could be used as a treatment for CRC. However, to date, no studies exploiting this alternative function have been reported. 

An interconnected pathway of regulation exists between melatonin, circadian clocks, and SIRT1 [27]. Agomelatine also regulates SIRT1 in several models of disease [28,29]. In this study, we evaluate the use of agomelatine as an antitumor therapy in established colorectal cell lines in vitro and in vivo in nude mice. We also analyze the implication of the SIRT1/PRX1 pathway in the regulation of the rhythmicity of core clock genes by agomelatine.

## 2. Materials and Methods

### 2.1. Cell Culture and Reagents

The colorectal adenocarcinoma cell lines HCT-116 (p53 wild type) and HCT-116 p53 null (Horizon Discovery Ltd., Cambridge, UK) were grown under standard conditions (37 °C and 5% CO_2_ in a humid atmosphere) using RPMI 1640 medium supplemented with 10% FBS and 1% penicillin-streptomycin (Gibco; Invitrogen, Carlsbad, CA, USA). For the experiments that required cell synchronization, cells were cultured in serum-free medium for 6 h prior to the initiation of the experiment [1]. Next, the cells were cultured in media with serum in the conditions required for the experiments carried out. 

Melatonin, agomelatine, fluorouracil (5-FU), and the remaining reagents employed in this study were obtained from Sigma-Aldrich (Sigma Chemical Co., St。 Louis, MO, USA).

### 2.2. Cell Viability Assay

Cell viability was analyzed with 3-(4,5-Dimethylthiazol-2-yl)-2,5-diphenyltetrazolium bromide (MTT) assay. Briefly, 4000 cells were seeded per well in a 96-well plate in a final volume of 100 μL. Twenty-four hours later, the cells were treated with different drugs and concentrations for 24, 48, and 72 h. Once the treatment was stopped, 10 μL of MTT (5 mg/mL) was added and the plate was incubated for 4 h at 37 °C and 5% CO_2_. Subsequently, 100 μL of lysis buffer (20% SDS in 50% formamide, pH 4.7) was added, and the plate was kept at 37 °C and 5% CO2 overnight. Optical density was measured with a Triad Multimode Microplate reader (Cultek SL, Madrid, Spain) at 570 nm.

### 2.3. Sphere-Formation Assay

Three-dimensional spheroids were generated and cultured in the InSphero GravityPLUS^TM^ Hanging Drop System (PerkinElmer), according to the manufacturer’s instructions. Briefly, cells were seeded at a density of 2500 cells per well in a 96-well GravityPLUS^TM^ Plate and placed in a 5% humidified CO_2_ incubator at 37 °C. After 3 days in culture, cells formed visible spheroids that were transferred to the GravityTRAP^TM^ Plate and allowed to grow for 4 more days. Every 2 days, the medium was changed. Subsequently, spheroids were treated with different doses of melatonin and agomelatine for 3 more days. Spheroids were imaged using a 4× objective on the image module of an EnSight^TM^ plate reader (PerkinElmer, Waltham, MA, USA). Acquired Images were automatically analyzed by the Kaleido 2.0 software.

### 2.4. Clonogenic Assay

Cells were seeded in 6-well plates at a concentration of 10^3^ cells/well and left to grow 72 h. Next, cells were treated for 72 h. Subsequently, the medium was changed, and cells were allowed to grow under standard conditions for 10 more days. To perform fixation and staining of colonies, the medium was removed, and the cells were incubated for 5 min with 0.5% crystal-violet-oxalate solution in 50% methanol. Colonies with more than 50 cells were counted.

### 2.5. Cell-Cycle Analysis

The percentage of cells in each cell-cycle phase was determined based on the cellular DNA content in at least 20,000 nuclei. Cells were seeded in 6-well plates and after treatments, they were harvested, washed with PBS, and fixed with 200 μL of 70% ice-cold ethanol at 4 °C for 30 min. Next, cells were washed with a solution of PBS containing 2% BSA and incubated in 500 μL of PI/RNase solution (Immunostep SL; Salamanca, Spain) at room temperature in darkness for 15 min. The percentages of cells in G0/G1, S, and G2/M phases were determined using a BD FACSAria IIIu flow cytometer (Becton Dickinson, BD Biosciences; Franklin Lakes, NJ, USA) from the Cytometry and Microscopy Research Service of the Biosanitary Research Institute of Granada. Experiments were performed at least three times and three samples per group were analyzed in each case.

### 2.6. Apoptosis Assay

The percentages of apoptotic cells in response to the different treatments were analyzed using a FITC Annexin V Apoptosis Detection Kit (BD Biosciences, Franklin Lakes, NJ, USA). Briefly, the cells were seeded in 6-well plates, and after treatments they were harvested and washed with PBS and concentrated at 1 × 10^6^ cells/mL. About 10^5^ cells (100 μL) were incubated with Annexin V-FITC and propidium iodide at room temperature and in darkness for 15 min. The samples were immediately analyzed using a BD FACSAria IIIu flow cytometer (Becton Dickinson, BD Biosciences; Franklin Lakes, NJ, USA) from the Cytometry and Microscopy Research Service of the Biosanitary Research Institute of Granada. The percentage of apoptosis was calculated by taking into account the sum of percentages of apoptotic cells (Annexin-FITC+/PI−) and late apoptotic cells (Annexin-FITC+/PI+).

### 2.7. Real-Time PCR

Total RNA was extracted using TRIzol reagent (Invitrogen, Carlsbad, CA, USA). The RNA was reverse-transcribed by RT-PCR into cDNA using the commercial kit, AccuScriptTM High Fidelity 1st Strand cDNA Synthesis Kit (Stratagene, Austin, TX, USA), according to the manufacturer’s instructions.

About 5 μL of the cDNA was amplified with specific primers for Per1, Per2, Per3, Cry1, Clock, Bmal1, Nr1d2, SIRT1, PRX1, and UBC (Table S1). Furthermore, PCR reactions with SYBER-green were performed using the Mx3000P qPCR System (Stratagene, Austin, TX, USA). Relative expression was calculated using UBC as a reference gene. Standard curves for each gene were made by plotting C_t_ values versus log cDNA dilution.

### 2.8. Immunoblot

Proteins were isolated from the lysed cells in RIPA buffer supplemented with protease inhibitors) for 30 min at 4 °C. The amount of proteins was quantified through Bradford assay, and samples were loaded in equal amounts (50 µg) into 12% SDS-polyacrylamide gels. The proteins resolved were transferred into PVDF-transfer membranes using a Bio-Rad Trans-Blot Turbo Transfer System (Bio-Rad Laboratories, Inc., Hercules, CA, USA). The blots were probed with the appropriate antibodies for caspase-3 (Santa Cruz Biotechnology, dilution 1:200), p53 (Santa Cruz Biotechnology, dilution 1:200), and ꞵ-Actin (Santa Cruz Biotechnology, dilution 1:200). As secondary antibody, HRP-conjugated anti-mouse antibody (Santa Cruz Biotechnology, dilution 1:50.000) was used. Amersham ECL Select Western Blotting Detection Reagent (GE Healthcare, Chicago, Il, USA) was applied before luminography for protein detection in a ChemiDoc MP System (Bio-Rad Inc., Hercules, CA, USA).

### 2.9. In Vivo Anti-Tumor Xenograft Studies

Female athymic Balb/c (nu/nu) mice (Charles Rivers Laboratory, Wilmington, MA, USA) housed in a regular 12:12 light–dark (LD) cycle (lights on at 08:00 h) were used for the in vivo studies. The mice were maintained in quarantine for a week before the subcutaneous injection of 2.5 × 10^6^ HCT-116 and HCT-116-p53-null cells suspended in 100 μL of RPMI 1640 medium into the right and left sides, respectively. Once the tumor size reached about 50–100 mm^3^, the animals were randomly divided into 4 groups (n = 8) and intraperitoneally treated for 2 weeks. Groups were as follows: (1) control (vehicle, three times per week); (2) melatonin (5 mg/kg, three times per week); (3) agomelatine (5 mg/kg, three times [er week); (4) 5-fluorouracil (50 mg/kg, two times per week). The agomelatine and melatonin groups were treated two hours before lights off, whereas the 5-fluorouracil group was treated 2 h after lights on. The tumor growth was measured three times per week and calculated by the formula V = (4π/3) × (width/2)2 × (length/2). Blood of each animal was extracted to analyze different biochemical parameters (glucose, urea, uric acid, AST, ALT, and amylase) by a Cobas c311 analyzer (Roche Diagnostics, Basel, Switzerland). Two weeks after the initiation of the treatments, the animals were sacrificed, and the tumors were extracted.

### 2.10. Statistical Analysis

All the experiments were performed at least in triplicate and data were expressed as mean ± SEM. To obtain circadian parameters, we determined the correct distribution (data interdependency and normal distribution) for each set of time-series data by lag plots/Q-test and normal probability plots/K-S test and, next, we calculated the acrophase and amplitude through the cosinor method using the TSA (Time Series Analysis—Cosinor 8.0 Lab View January 2020) software (http://www.euroestech.com/, accessed on 21 January 2020). Detection of rhythm was achieved by rejection of the zero-amplitude hypothesis with 95% certainty, as reflected by the *p* value. The amplitude (i.e., the difference between the peak or trough and the mean value of a cosine curve), acrophase (i.e., the phase angle of the peak of a cosine curve), and midline estimating statistic of rhythm (MESOR) (i.e., the average value of cosine curve fitted to the data) were compared where applicable using 48-h trial period, since, in some cases, the oscillation period of the genes studied was close to this time. Nevertheless, comparisons were also performed using a 24-h test period (Tables S2–S5), with similar results obtained at both periods. Data from fitted curves were transferred to GraphPad Prism (GraphPad, La Jolla, SD, USA). Rhythm characteristics (MESOR, amplitude, acrophase) for each variable were compared by a non-parametric test [30,31].

## 3. Results

### 3.1. Agomelatine Inibits the Growth of Human CRC Cells in a p53-Dependent Manner

**Figure 1:**
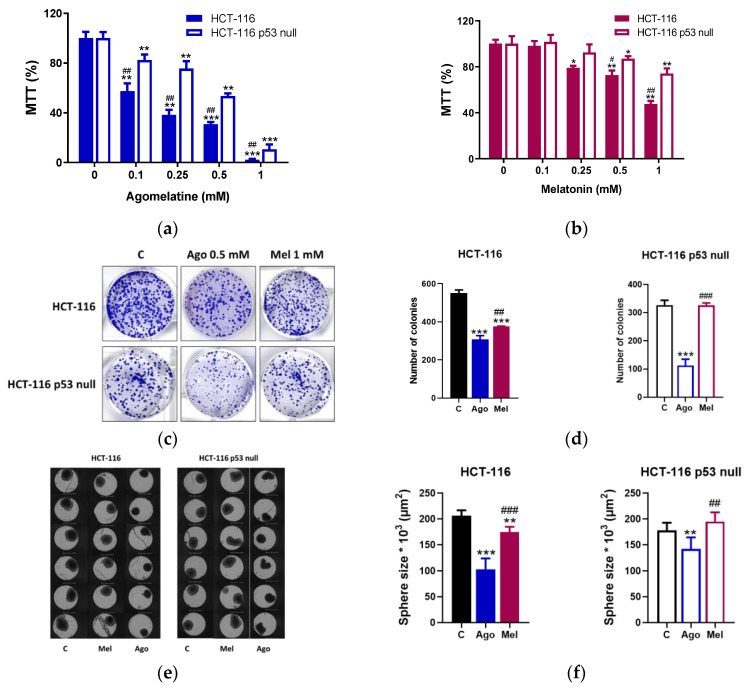
Inhibition of viability on HCT-116 and HCT-116-p53-null cells by (**a**) agomelatine and (**b**) melatonin. The results represent the mean ± SD of three experiments performed in quadruplicate. * *p* < 0.05 vs. C; ** *p* < 0.01 vs. C; *** *p* < 0.001 vs. C; ^##^ *p* < 0.01 vs. the same concentration of agomelatine (Ago) or melatonin (Mel); ^#^ *p* < 0.05 vs. the same concentration of melatonin (Mel) or agomelatine (Ago). (**c**) Representative example of colony-formation assay and (**d**) graphical representation of three experiments in HCT-116 and HCT-116-p53-null cells after treatment with agomelatine (Ago) (0.5 mM) and melatonin (Mel) (1 mM) versus control (C) cells. Results are presented as means ± SD. *** Values of *p* < 0.001 versus C; ^##^ *p* < 0.01 vs. agomelatine; ^###^ *p* < 0.001 vs. agomelatine. In other experiments, (**e**) HTC-116 and HCT-116-p53-null cells were cultured in a 3D model and (**f**) the size of the spheroids was measured in control and after agomelatine (Ago) (0.5 mM) and melatonin (Mel) (1 mM) treatments during 72 h. ** Values of *p* < 0.01 vs. C; *** *p* < 0.001 vs. C; ^##^ *p* < 0.01 vs. melatonin; ^###^ *p* < 0.001 vs. melatonin.

Two established CRC cell lines, HCT-116 (wild-type p53) and HCT-116 p53 null, were used to evaluate the antiproliferative effects of agomelatine in vitro. Cells were cultured in the presence of increasing concentrations of agomelatine (0–1 mM) for 24, 48, and 72 h. Treatment of both cell lines with agomelatine led to a dose-dependent inhibitory effect on cell growth at all times studied (Figure S1a,b). Similarly, the effect of melatonin was dose-dependent in both cell lines, although in HCT-116-p53-null cells, the indoleamine reduced cell growth significantly after 48 h treatment and at the highest dose studied (1 mM) (Figure S1c,d). We next compared the effects of both drugs regarding the p53 status of the cells at 72 h. Treatment of both cells lines with agomelatine inhibited cell growth significantly from the lowest dose used (0.1 mM), although the effect was higher in the HCT-116 than in the HCT-116 p53 null (Figure 1a). As we expected, melatonin was more effective on the HCT-116 than on the HCT-116 p53 null. As shown in Figure 1b, melatonin significantly inhibited the HCT-116’s growth from 0.25 mM, whereas in the HCT-116 p53 null, the effect was statistically significant from 0.5 mM. These results indicate that agomelatine is more potent than melatonin in inhibiting CRC growth in vitro. 

We also conducted a clonogenic assay for up to 10 days after treatment to evaluate the long-term effects of agomelatine and melatonin on the cancer-cell survival (Figure 1c). Notably, the number of colonies in the HCT-116 cells reduced after the treatments with agomelatine (0.5 mM) and melatonin (1 mM), although the effect of agomelatine was significantly higher (*p* < 0.01) (Figure 1d). Only agomelatine (0.5 mM) was able to reduce the number of colonies in the HCT-116-p53-null cells (Figure 1d). 

To corroborate these results, we also performed a 3D-culture model. To identify the sizes of the spheroids formed (Figure 1e), images were obtained and, subsequently, the perimeter was calculated (Figure 1f). According to the results described in the 2D model, both agomelatine (0.5 mM) and the melatonin (1 mM) reduced the sizes of the spheroids in the HCT-116, and agomelatine was more potent than melatonin (*p* < 0.001) at the doses used. In the HCT-116-p53-null cells, only agomelatine was able to reduce the sizes of the spheroids formed.

### 3.2. Agomelatine Induces Cell-Cycle Arrest and Caspase-Dependent Apoptosis in CRC Cells

**Figure 2:**
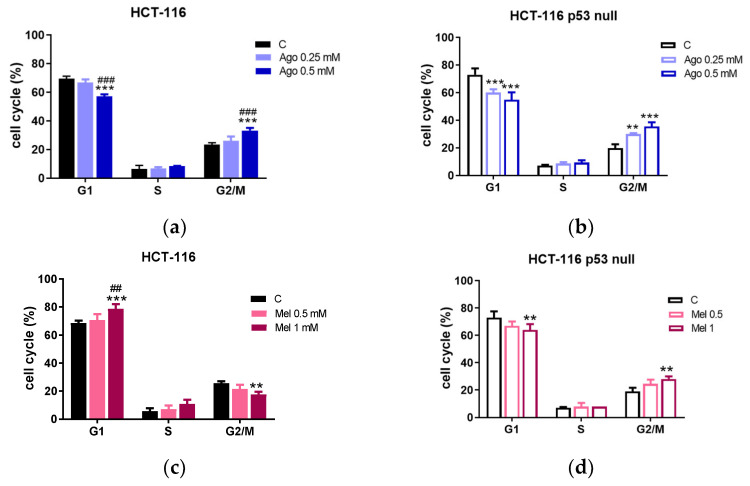
Percentage distribution in the different stages of the cell-cycle after treatment of HCT-116 and HCT-116-p53-null cells with agomelatine (Ago) (**a**,**b**) and melatonin (Mel) (**c**,**d**). Data represent the mean ± SD of three experiments performed in triplicate. ** Values of *p* < 0.01 vs. C; *** *p* < 0.001 vs. C. ^##^ *p* < 0.01 vs. lower doses of treatment; ^###^ *p* < 0.001 vs. lower doses of treatment.

The cell-cycle distribution after agomelatine and melatonin treatments was assessed by flow cytometry (Figures S2 and S3). The treatment with 0.5 mM of agomelatine for 72 h induced accumulation in the G2/M phase of the cycle in the HCT-116 (33.25 ± 1.91% of agomelatine-treated cells in the G2/M vs. 23.35 ± 1.42% of control cells in the same phase) and the HCT-116 p53 null (35.50 ± 1.13% of agomelatine-treated cells in the G2/M vs. 19.90 ± 1.42% of control cells in the same phase). Agomelatine treatment also resulted in a decrease in the number of cells in the G1 phase in the HCT-116 (33.25 ± 5.24% in the agomelatine-treated group vs. 69.47 ± 1.72% in the control cells) and HCT-116 p53 null (54.90 ± 2.07% in the agomelatine-treated group vs. 73.00 ± 2.68% in the control cells). The treatment of the cells with 0.25 mM agomelatine produced similar variations in cell-cycle distribution in the HCT-116-p53-null cells (Figure 2a,b). 

The treatment with 1 mM melatonin for 72 h induced accumulation in the G1 phase of the cycle in the HCT-116 (78.70 ± 3.33% of melatonin-treated cells versus 68.5 ± 1.83% of control cells). This was associated with a decrease in the number of cells in the G2/M (17.70 ± 1.91% in the melatonin-treated group vs. 25.60 ± 1.42% in control cells). In the HCT-116-p53-null cells, we found an accumulation of cells in the G2/M phase (28.10 ± 1.91% of melatonin-treated cells versus 19.00 ± 2.73% of control cells) and a decrease in the G1 phase (64.00 ± 4.33% in the melatonin-treated group vs. 73.00 ± 4.59% in the control cells). The treatment with 0.5 mM melatonin did not produce any significant variations in the cell-cycle distribution in either of the two lines tested (Figure 2c,d).

In addition to the effects on the cell growth and cell cycle in vitro, we studied whether agomelatine and melatonin induced cell death through apoptosis using annexin V and propidium iodide and analyzing cell populations stained by flow cytometry (Figures S4 and S5). 

**Figure 3:**
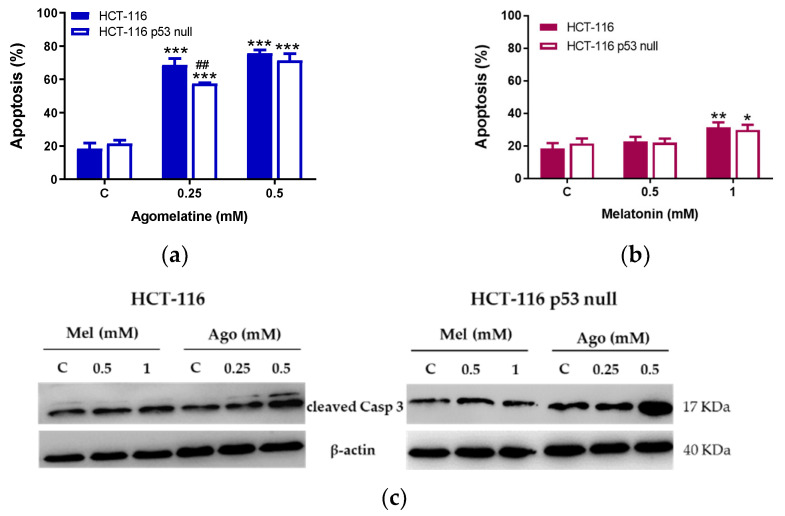
Percentage of apoptosis in HCT-116 and HCT-116-p53-null cells after agomelatine (**a**) and melatonin (**b**) treatments. Data represent the mean ± SD of three experiments performed in duplicate. * Values of *p* < 0.05 vs. C; ** *p* < 0.01 vs. C; *** *p* < 0.001 vs. C; ^##^ *p* < 0.01 vs. HCT-116. (**c**) Expression of cleaved caspase-3 after treatment for 72 h with different doses of melatonin (Mel) and agomelatine (Ago) on HCT-116 and HCT-116 p53 null.

The treatment with agomelatine induced a high percentage of apoptosis in both cell lines after 72 h at the doses studied (Figure 3a). Differences were found in the number of apoptotic cells in the two lines, although they were only observed at the lowest dose of agomelatine used (0.25 mM), and were greater in the HCT-116 cells (Figure 3a). When we used melatonin as a treatment for 72 h, we found increased apoptosis in the two cell lines tested only at 1 mM, while a smaller concentration of the indolamine (0.5 mM) did not produce cytotoxicity in either of the two lines analyzed. As expected, the percentage of cell death by apoptosis was higher in both cell lines treated with agomelatine.

As shown in Figure 3c, the cell death induced by agomelatine was caspase-dependent, since this treatment induced increased the expression of active caspase-3 (cleaved caspase-3), at least at the higher doses used (0.5 mM), in the HCT-116 and HCT-116-null cells.

### 3.3. Regulation of Tumor Growth In Vivo by Agomelatine

To study their effect on tumor growth in vivo and to analyze possible side effects after treatment with both drugs, the HCT-116 and HCT-116-p53-null cell lines were injected into the right and left flanks of the immunosuppressed Balb/c nu/nu mice, respectively. Agomelatine and melatonin were injected at a dose of 5 mg/kg of body weight three times per week. A group of animals injected with 5-fluorouracil (5-FU) at a dose of 50 mg/kg twice per week was also used. This treatment was chosen to compare the effects of both agomelatine and melatonin, since it remains the first-line treatment for CRC, both alone (capecitabine) or in combination with other drugs (oxaliplatin and irinotecan, among others). The protein p53 serves as the major route for the anti-cancer effect of 5-FU and determines the cellular sensitivity to cytotoxic 5-FU [32] In fact, the absence of an active p53 drastically reduces its effectiveness [33].

**Figure 4:**
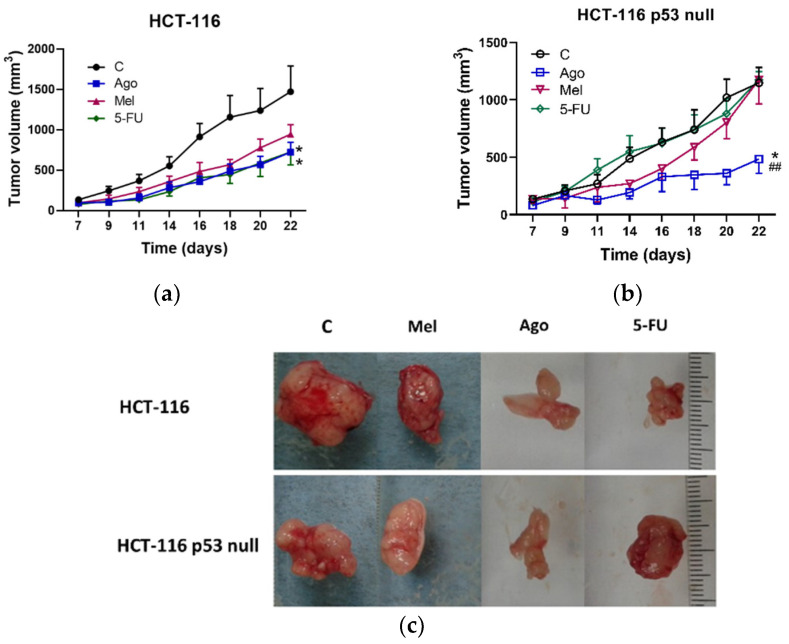
Tumor growth in the four groups of nude mice (controls and those treated with agomelatine, melatonin, or 5-FU, as described in Materials and Methods) in cell-line-derived xenografts in either (**a**) HCT-116 or (**b**) HCT-116 p53 null. Data represent mean ± SEM. * *p* < 0.05 vs. control mice; ^##^ *p* < 0.01 vs. 5-FU group. (**c**) Representative images of tumors in control and treated mice in cell-line-derived xenografts. C: control; Mel: melatonin; Ago: agomelatine; 5-FU: 5-fluorouracil.

At the doses used, all the drugs reduced the sizes of the tumors generated by the HCT-116 cells. In this case, agomelatine and 5-FU showed similar levels of potency (*p* < 0.05 vs. non-treated mice). Melatonin showed an almost statistically significant effect (*p* = 0.076) (Figure 4a). In the tumors generated by the HCT-116-p53-null cells, only agomelatine was able to reduce their size (*p* < 0.05), and its effect was significantly different from that of the 5-FU (*p* < 0.01) (Figure 4b). At the systemic level, agomelatine and melatonin led to an increase in blood glucose and alanine transaminase (ALT), whereas the treatment with 5-FU induced an increase in ALT levels (Figure S6).

### 3.4. Agomelatine Regulates p53-Protein Levels in In Vitro and In Vivo Models of CRC

**Figure 5:**
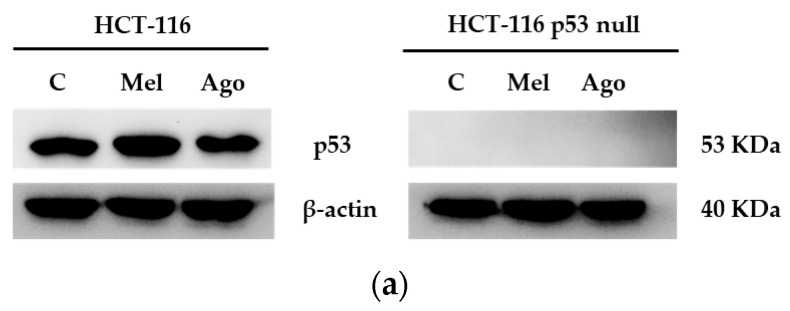

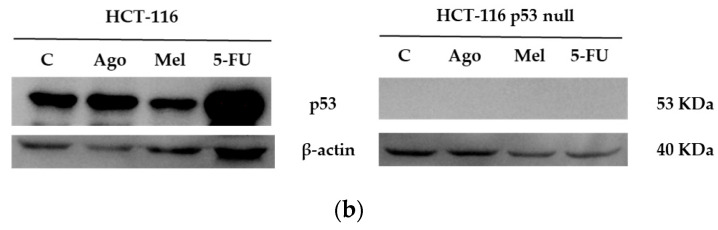
Expression levels of p53-protein in response to the treatments (**a**) with 0.5 mM agomelatine (Ago) and 1 mM melatonin (Mel) in the in vitro model, and (**b**) with agomelatine (Ago), melatonin (Mel), and 5-5-fluorouracil (FU) in tumors derived from HCT-116 and HCT-116-p53-null cell lines.

Melatonin and 5-FU regulate the expression of p53 protein levels and, as mentioned above, the presence of p53 is crucial for 5-FU’s effectiveness [32,33,34]. As an analogue of melatonin, we analyzed whether agomelatine also regulated the levels of p53 in our in vitro and in vivo models. As shown in Figure 5a, agomelatine induced a small increase in the expression of the p53 protein after 72 h of treatment in the HCT-116 cells, although no effect was found after melatonin treatment at this point. Similar results were found in the in vivo model, in which the 5-FU displayed the highest effect (Figure 5b). As expected, the expression of the p53 protein was absent in the in vitro and in vivo models derived from the HCT-116-p53-null line (Figure 5).

### 3.5. Agomelatine Regulates Circadian-Clock Genes’ Rhythmicity in CRC Cell Lines In Vitro

**Figure 6:**
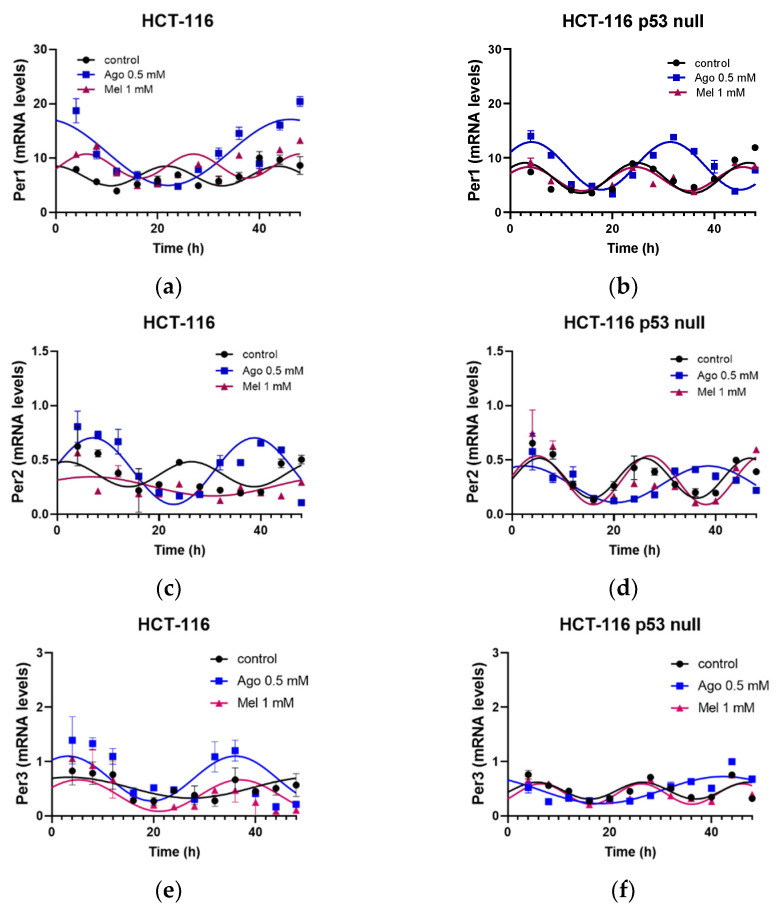

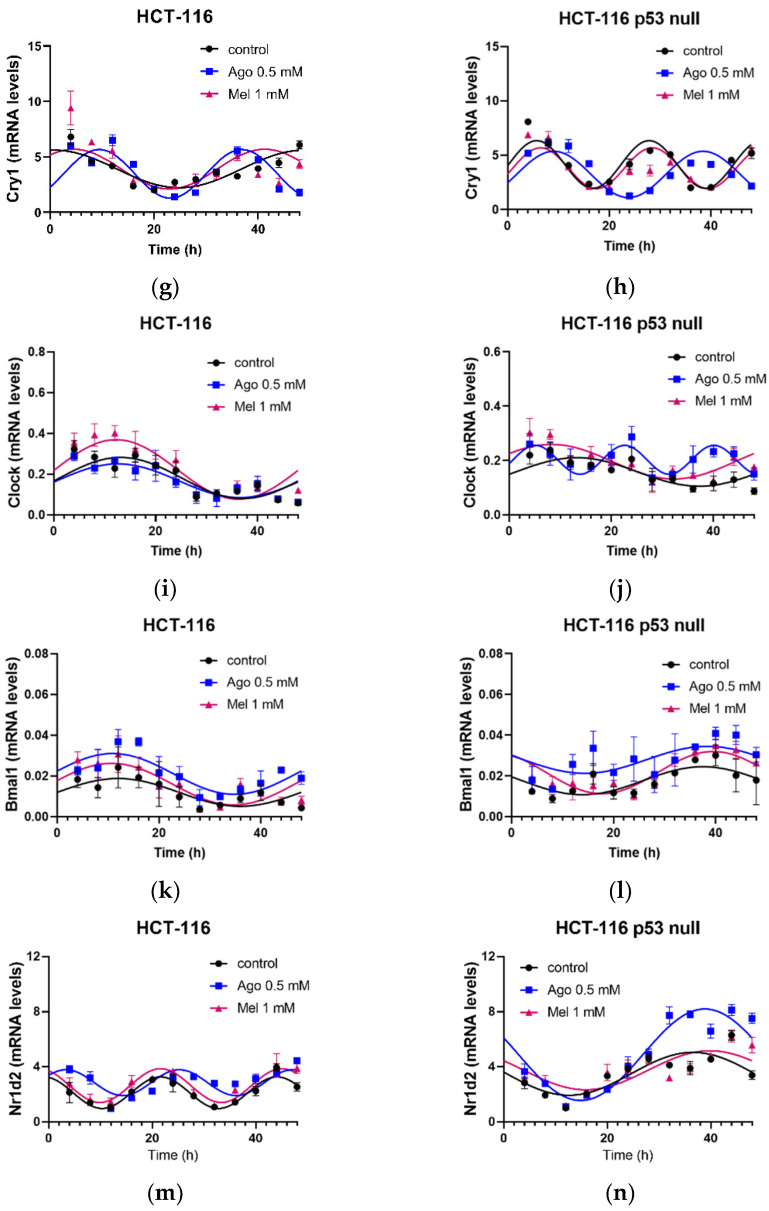
Characteristics of the rhythms obtained for Period 1 (Per1) (**a**,**b**), Period 2 (Per2) (**c**,**d**), Period 3 (Per3) (**e**,**f**), cryptocrome1 (Cry1) (**g**,**h**), circadian locomotor cycles kaput (Clock) (**i**,**j**), brain and muscle ARNT-like (Bmal1) (**k**,**l**) and nuclear receptor subfamily 1 group D member 2 (Nr1d2) (**m**,**n**) gene after agomelatine (Ago) and melatonin (Mel) treatments in the HCT-116 and HCT-116-p53-null cell lines. Curve fittings under the different conditions were performed using a 48-h test period.

Next, we analyzed the involvement of circadian-clock genes in the effects on the cell growth and viability induced by agomelatine and melatonin in both cell lines. Given that the effect of 0.5 mM agomelatine on cell growth was significant from 24 h in both cell lines, after cell-culture synchronization by serum shock for 6 h, the cells were cultured in the presence of 10% FBS and treated with vehicle, 0.5 mM agomelatine, or 1 mM melatonin for 48 h. The expressions of Per1-3, Cry1, Clock, Bmal1, and Nr1d2 were analyzed every 4 h (Figure 6). 

We found a statistically significant rhythmicity in all the genes studied in the HCT-116 and HCT-116-p53-null cells (Table 1 and Table 2). However, these cells showed some differences in the characteristics of their rhythmicity (Table 2). The acrophase of the Per2, Cry1, Bmal1, and Nr1d2 showed an advance in phase in the HCT-116-p53-null cells versus their isogenic HCT-116 cells. The MESOR levels of the Per1 and Per3 were lower in the HCT-116-p53-null cells than in the HCT-116 cells, but this parameter of Bmal1 was higher in the cell line HCT-116 p53 null. There were also differences in the amplitudes of some of the genes between these cells, although these were smaller than those found for the acrophase and the MESOR. The Per2 and Nr1d2 showed lower amplitudes in the HCT-116-p53-null cells than in the HCT-116, while this parameter was higher for the Bmal1.

The treatments with agomelatine and melatonin in the HCT-116 control cells maintained the rhythmicity of the genes studied, albeit with some differences (Table 1). Both drugs induced an advance in phase in the acrophase of the Per1, Per2, Per3, and Cry1; however, the effect of agomelatine was significantly higher in all these genes, except in the case of the Per3, in which both treatments had a similar effect. Only agomelatine induced a delay in the acrophase of the Nr1d2. No significant effects were found on the acrophase in the Clock or Bmal1. Agomelatine increased the MESOR in the Bmal1 and decreased it in the Nr1d2, while melatonin increased this parameter in the Clock. Both treatments increased the amplitude in the Per3. Agomelatine increased the amplitude in the Per2 and decreased it in the Nr1d2. Melatonin treatment decreased the amplitude in Per1 compared with the agomelatine treatment, while increased it in the Clock.

Similar to the results found in the HCT-116 cells, both treatments maintained the rhythmicity of the genes analyzed in the HCT-116-p53-null cells (Table 2). In these cells, only agomelatine was able to induce an advance in phase in the acrophase of the Per1, Per3, and Cry1 compared with the non-treated cells, whereas Per2 and Clock acrophase showed a delay in phase. Agomelatine also increased the MESOR in the Per1, Clock, and Nr1d2, while it decreased the MESOR in the Cry1. Melatonin also enhanced the MESOR in the Clock and reduced it in the Bmal1. Agomelatine increased the amplitude in the Per3 and Nr1d2. Melatonin did not change this parameter in any of the genes studied in this cell line.

**Figure 7:**
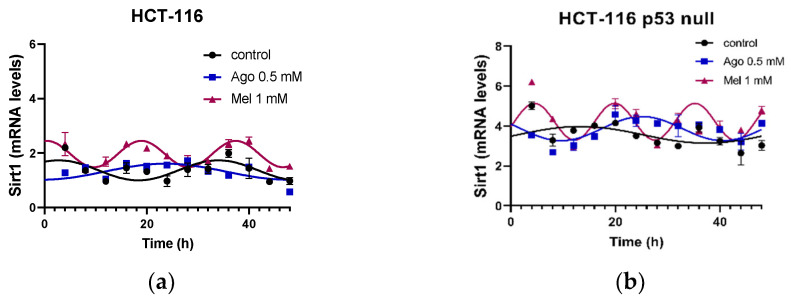
Characteristics of each of the rhythms obtained for sirtuin 1 (SIRT1) gene after agomelatine (Ago) and melatonin (Mel) treatments in the HCT-116 (**a**) and HCT-116-p53-null (**b**) cell lines. Curve fittings under the different conditions were performed using a 48-h test period.

We also analyzed the rhythmicity of the Sirt1 in the non-treated cells and after the agomelatine and melatonin treatments in both cell lines (Figure 7). The HCT-116 cells showed a delay in phase in the acrophase and lower MESOR levels than the HCT-116-p53-null cells (Table 3). Agomelatine and melatonin changed the acrophase of the genes in both types of cell, but while agomelatine induced an advance in phase of this parameter, melatonin delayed it. Melatonin also increased the amplitude of the SIRT1 rhythmicity. 

**Figure 8:**
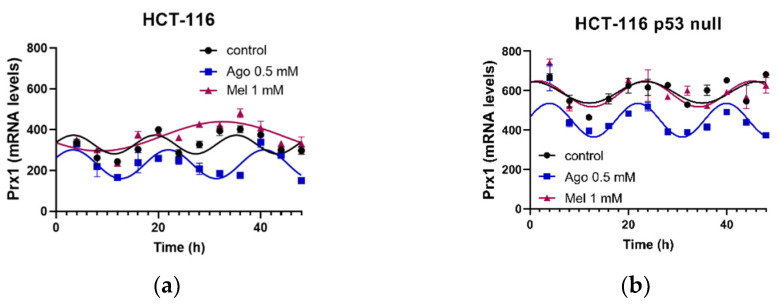
Circadian rhythms obtained for peroxiredoxin 1 (PRX1) gene after agomelatine (Ago) and melatonin (Mel) treatments in the HCT-116 (**a**) and HCT-116-p53-null (**b**) cell lines. Curve fittings under the different conditions were performed using a 48-h test period.

In order to find differences between the mechanisms of action of agomelatine and melatonin that could explain the differences in rhythmicity found between the genes studied in the two cell lines, we also analyzed the PRX1 in the non-treated cells and after the agomelatine and melatonin treatments (Figure 8). The PRX1 acrophase in the HCT-116 cells showed an advance in phase but lower MESOR than in the HCT-116-p53-null cells (Table 4). Agomelatine and melatonin induced changes in different parameters in the HCT-116 cells (Figure 8a). Melatonin induced an advance in phase in the acrophase and the MESOR of the gene, while agomelatine delayed this parameter. By contrast, agomelatine induced an advance in phase in the acrophase and decreased the MESOR of the PRX1 in the HCT-116-p53-null cells (Figure 8b), while melatonin did not induce changes in any of the rhythmic parameters in this gene (Table 4). 

## 4. Discussion

This study describes, for the first time, the potential use of agomelatine as a treatment for CRC. Agomelatine inhibited cell growth in our in vitro and in vivo models of this disease and induced cell-cycle arrest and caspase-dependent apoptotic cell death. We propose that agomelatine exerted these effects through the regulation of the rhythmicity of several core Clock genes. The effect of agomelatine was independent of the p53, although this treatment decreased the cell growth more efficiently in the in vitro models of cells harboring the wild-type p53. 

Similarly, melatonin induced cell-growth inhibition in vitro and in vivo and cell-cycle arrest. However, it was not able to increase apoptosis, as expected. These effects were only observed in the presence of the wild-type p53, since p53 activation is critical for the oncostatic effect of melatonin, encouraging p53 accumulation at the cellular level [34]. However, the expression of the protein increases transiently, displaying a maximum at three hours after the treatment of with melatonin cells [34]. This could explain the lack of effect found in our study, in which the effect on the p53-protein levels was assessed after 72 h of melatonin treatment. The inhibition of tumor growth after the treatment with melatonin was related to the inhibition of cell progression from the G0/G1 phase to the S phase. This effect was dependent on the p53 status, since melatonin induces the activation of p53 and p21. The observed increase in the percentage of apoptosis after the treatment with the indoleamine may have been due to the blockade of the Akt/MDM2 pathway, an effect that was previously observed in gastric cancer cells [35]. Increased apoptosis and cell-cycle arrest should trigger the activation of the caspase-3 pathway, as described for hepatocarcinoma cells [36]. Considering these observations, in our model, the fact that we did not observe an increase in caspase-3 expression after melatonin treatment may have been due to the small increase in apoptosis we obtained. 

Intracellular melatonin signaling through its receptors can also be affected by the status of p53. It has been reported that the expression of the membrane (MT1 and MT2) receptors of melatonin decreases in patients with colon cancer [22], but mainly in those with mutations in the p53 gene [23], although melatonin levels and the expression of nuclear (RORα) receptors do not change [22]. On the other hand, several works previously showed the implication of melatonin receptors in its oncostatic actions in this disease [37,38]. This could indicate that non-selective MT1/MT2 agonists with a higher affinity with these receptors than melatonin itself could be of interest in the treatment of CRC [22]. In line with this observation, we found that agomelatine was more potent than melatonin, at least in the in vitro and in vivo models in which the tumor-suppressor gene p53 was in its wild form. In this case, agomelatine seemed to induce a slight increase in the p53 protein levels at the time at which it was analyzed. In addition, agomelatine was more potent than the indoleamine in inhibiting cell growth in the in vitro models with the non-active p53. Interestingly, unlike melatonin, as described above, agomelatine inhibited tumor growth in vivo in the cases with the non-functional p53, indicating that mechanisms other than p53 regulation may be involved in its mechanism of action, at least under these conditions. Agomelatine has been shown to be more effective than melatonin when used as a treatment for insomnia, depression, or obesity-associated comorbidities [39,40,41]. Agomelatine is a non-selective agonist of MT1 and MT2 receptors, with a higher affinity with these receptors than melatonin itself [42]. In addition, agomelatine has a longer half-life and better oral absorption [43]. On the other hand, agomelatine is a serotonin-HT-2c- and -HT-2b-receptor antagonist. The involvement of serotonin in tumor growth, differentiation, and gene expression has long been known. In fact, its mitogenic effect has been demonstrated in various types of cancer, including prostate, bladder, breast, and colon cancer [44]. In the specific case of colon cancer, selective serotonin reuptake inhibitors (SSRIs), which increase its concentration in nerve terminals both centrally and peripherally, have been shown to have preventive effects [45]. 

In our in vitro model, we found the resynchronization of circadian rhythmicity by agomelatine and melatonin in vitro. Both drugs regulated Per1, Per2, Per3, Cry1, and Clock in cells harboring the wild-type p53. The change induced in the characteristics of the rhythms analyzed (i.e., amplitude, acrophase, and/or MESOR) was always higher after the treatment with agomelatine. In these cells, agomelatine also regulated Bmal1 and Nr1d2. The deletion of p53 induced a loss of effect in both drugs, although agomelatine was also found to be more potent than melatonin. In this type of cell, we also found a decrease in the number of circadian genes regulated by both drugs, indicating the influence of the circadian clock on the antiproliferative effect of these treatments. Previous reports showed that melatonin regulates the circadian clock after the activation of MT1 and MT2 receptors at the central level [39] and in cancer [46], whereas agomelatine acts at the central level in a complementary and possibly synergistic manner on the MT1 and MT2 receptors of melatonin and the HT2c receptor of serotonin to resynchronize circadian rhythms [47,48], which could explain the greater effect found after the treatment with agomelatine.

The SIRT1 is a NAD+ dependent class III histone deacetylase, which is involved in the control of cellular redox homeostasis [6]. It regulates the circadian clock by activating the transcription of BMAL1 and Clock [6], and the intracellular levels of SIRT1 and NAD+ oscillate with a circadian pattern [7]. Although the exact role of SIRT1 is not well understood, it has been implicated in tumor growth and resistance to therapy in several types of cancer, including CRC [49,50]. An important relationship between melatonin, the circadian clock, and SIRT1 has been described, although it remains unclear whether SIRT1 is a key mediator of circadian-clock regulation by melatonin or whether melatonin acts through circadian genes to regulate SIRT1 [51]. Peroxiredoxins (PRX) are a family of conserved enzymes involved in the regulation of peroxide levels. The oxidation-reduction states of PRX1 proteins exhibit self-sustained oscillation in the absence of TTFL mechanisms. In addition, the knockdown of PRX1 proteins affects circadian rhythms in nucleated cells, indicating that the oxidation-reduction cycle of PRX1 is interconnected with the main circadian-clock cycle [52]. Furthermore, Tp53 and SIRT1 are mediators of these connections [52], which are important regulators of circadian-clock-gene expression [8,53,54]. In fact, in the present study, we found that agomelatine and melatonin can regulate the rhythmicity of SIRT1, regardless of p53, although they differ in the characteristics of the rhythmicity affected. Interestingly, only agomelatine was able to regulate the oscillations of the PRX1 in both the wild-type p53 and p53 null cells, whereas melatonin only affected it in the wild-type p53 cells. Further research is warranted to determine the mechanism underlying these differences. The antioxidant ability of melatonin is extended to the regulation of the expression and activity of PRX1 [55], although there are no available data regarding the role of p53 in this action of the indoleamine.

The antitumor efficacy of 5-FU is due to its ability to induce cell-cycle arrest and p53-dependent apoptosis [56]. Similarly to melatonin, several lines of research demonstrated that this chemotherapeutic agent has a reduced ability to inhibit cell growth in CRC with mutated or inactive p53 [57]. In this study, we used the treatment with 5-FU as a control for the in vivo model carrying p53 null tumors, and only agomelatine was able to reduce tumor growth in these animals. Although the side effects found at the systemic level were similar after the three treatments, the 5-FU induced the greatest increase in AST activity. At the metabolic level, melatonin treatment produced an increase in glucose and alanine-aminotransferase levels, probably due to the role of melatonin in processes such as glycogenesis or glycolysis [36].

## 5. Conclusions

Recent studies have shown that some anti-depressant treatments also exert an anti-tumor effect, which is due to the modification of the tumor environment or the alteration of the immune response. This is the case in colon-cancer , in which the use of fluoxetine inhibits the transcriptional activity of NF-kappa B, and the proliferation of cells [58]. In addition, in general, the use of these drugs does not present a large number of side effects in the organism [59]. Therefore, taking into account the fact that treatment with antidepressant drugs after a cancer diagnosis is quite frequent, if it is possible to perform both treatments with the same drug, as is the case with agomelatine, conferring a double benefit. In addition, the presence of a non-functional p53 has been implicated in 5-FU resistance in CRC patients [57]. This barrier could be overcome with treatment with agomelatine, since it has similar effectiveness regardless of p53 status, at least in the xenograft model used in this study.

## Figures and Tables

**Table 1:** Results of the cosinor analysis of the clock genes’ expression after agomelatine or melatonin treatment in the HCT-116 cell line.

Gene	Treatment	PR ^1^	*p*-Value ^2^	Amplitude (A.U.) ^3^	Acrophase (h) ^4^	MESOR (A.U.)
Per1	Control	29.41	0.03	5.17 ± 1.45	3.06 ± 1.91	9.77 ± 0.99
	Ago 0.5	42.85	0.003	6.06 ± 1.22	45.30 ± 1.53 ***	10.40 ± 0.86
	Mel 1	34.77	0.008	2.24 ± 0.53 ^#^	32.83 ± 2.00 ***^,###^	8.25 ± 0.39
Per2	Control	31.66	0.006	0.076 ± 0.018	2.76 ± 1.19	0.28 ± 0.01
	Ago 0.5	42.02	0.001	0.140 ± 0.003 *	9.35 ± 0.08 ***	0.25 ± 0.02
	Mel 1	27.81	0.003	0.088 ± 0.024	6.58 ± 2.09 ***^,###^	0.26 ± 0.02
Per3	Control	25.36	0.008	0.33 ± 0.09	17.47 ± 1.15	0.72 ± 0.07
	Ago 0.5	60.62	0.0001	0.91 ± 0.14 *	6.06 ± 0.68 ***	0.94 ± 0.09
	Mel 1	31.33	0.002	0.96 ± 0.25 *	5.55 ± 1.44 ***	0.83 ± 0.18
Cry1	Control	63.01	<0.0001	1.62 ± 0.22	1.79 ± 1.02	3.81 ± 0.15
	Ago 0.5	73.64	<0.0001	2.19 ± 0.21	9.47 ± 0.48 ***	3.48 ± 0.16
	Mel 1	37.72	0.0004	1.79 ± 0.42	4.55 ± 1.31 **^,###^	3.90 ± 0.29
Clock	Control	57.99	<0.0001	0.101 ± 0.015	13.14 ± 1.13	0.18 ± 0.01
	Ago 0.5	56.69	0.001	0.111 ± 0.019	11.76 ± 2.97	0.16 ± 0.01
	Mel 1	56.75	<0.0001	0.189 ± 0.029 **^,###^	10.77 ± 1.16	0.26 ± 0.02 ***^,###^
Bmal1	Control	61.01	<0.0001	0.0088 ± 0.0012	12.99 ± 1.06	0.014 ± 0.001
	Ago 0.5	71.30	<0.0001	0.0138 ± 0.0015	10.47 ± 0.84	0.025 ± 0.001 ***
	Mel 1	38.63	0.0003	0.0092 ± 0.0020	10.85 ± 1.67	0.016 ± 0.001 ^###^
Nr1d2	Control	48.84	<0.0001	1.73 ± 0.32	12.19 ± 0.63	3.79 ± 0.22
	Ago 0.5	55.56	<0.0001	0.94 ± 0.15 **	3.32 ± 0.55 ***	2.86 ± 0.11 ***
	Mel 1	57.84	<0.0001	1.89 ± 0.28 ^##^	11.55 ± 0.56 ^###^	3.74 ± 0.20 ^###^

^1^ PR: percentage of rhythm; ^2^ *p*-value: zero-amplitude test; ^3^ A.U.: arbitrary units; ^4^ h: hours. Comparisons of parameters under the different conditions were performed using a 48-h test period. * Values of *p* < 0.05 vs. control, ** *p* < 0.01 vs. control, *** *p* < 0.001 vs. control; ^#^ *p* < 0.05 vs. agomelatine, ^##^ *p* < 0.01 vs. agomelatine, ^###^ *p* < 0.001 vs. agomelatine.

**Table 2:** Results of the cosinor analysis of the clock genes’ expression after agomelatine or melatonin treatment in the HCT-116-p53-null cell line.

Gene	Treatment	PR ^1^	*p*-Value ^2^	Amplitude (A.U.) ^3^	Acrophase (h) ^4^	MESOR (A.U.)
Per1	Control	47.47	0.0001	2.42 ± 0.45	3.01 ± 0.65	6.11 ± 0.33 ^†^
	Ago 0.5	51.58	0.003	2.54 ± 1.22	4.80 ± 3.68 ***	8.36 ± 0.86 **
	Mel1	68.24	<0.0001	2.2 ± 0.26	2.61 ± 0.41 ^###^	6.13 ± 0.19 ^##^
Per2	Control	61.90	<0.0001	0.22 ± 0.03^†^	4.89 ± 0.48 ^†††^	0.34 ± 0.02
	Ago 0.5	44.57	0.0001	0.19 ± 0.04	1.29 ± 1.16 ***	0.29 ± 0.03
	Mel 1	56.40	<0.0001	0.25 ± 0.04	5.44 ± 0.56 ^###^	0.32 ± 0.03
Per3	Control	40.65	0.0002	0.13 ± 0.03	6.02 ± 0.71	0.37 ± 0.02 ^††^
	Ago 0.5	65.87	<0.0001	0.21 ± 0.03 *	42.48 ± 0.95 ***	0.37 ± 0.02
	Mel 1	56.89	<0.0001	0.17 ± 0.02	6.41 ± 1.16 ^###^	0.34 ± 0.02
Cry1	Control	78.47	<0.0001	2.19 ± 0.20	5.73 ± 0.34 ^†††^	4.16 ± 0.14
	Ago 0.5	79.43	<0.0001	2.13 ± 0.17	9.11 ± 0.42 *	3.25 ± 0.13 ***
	Mel 1	67.89	<0.0001	1.88 ± 0.22	6.51 ± 0.44	3.82 ± 0.16 ^##^
Clock	Control	41.13	0.0002	0.05 ± 0.01 ^†^	11.99 ± 1.59	0.16 ± 0.01
	Ago 0.5	73.01	0.0001	0.04 ± 0.01	4.57 ± 0.76 ***	0.19 ± 0.01 **
	Mel 1	56.47	<0.0001	0.06 ± 0.01	8.86 ± 1.17 ^##^	0.19 ± 0.01 **
Bmal1	Control	58.43	<0.0001	0.018 ± 0.003 ^†††^	35.12 ± 1.09 ^†††^	0.025 ± 0.002 ^†††^
	Ago 0.5	50.78	<0.0001	0.011 ± 0.002	39.95 ± 1.31	0.022 ± 0.001
	Mel 1	37.40	0.0004	0.010 ± 0.002	37.92 ± 1.56	0.020 ± 0.002 *
Nr1d2	Control	45.84	<0.0001	1.41 ± 0.27 ^††^	35.29 ± 1.44 ^†††^	3.25 ± 0.19
	Ago 0.5	71.46	<0.0001	3.78 ± 0.41 ***	37.66 ± 0.84	5.54 ± 0.29 ***
	Mel 1	44.92	0.0001	2.08 ± 0.40 ^##^	36.94 ± 1.47	4.07 ± 0.28 ^###^

^1^ PR: percentage of rhythm; ^2^ *p*-value: zero-amplitude test; ^3^ A.U.: arbitrary units; ^4^ h: hours. Comparisons of parameters at the different conditions were performed using a 48-h test period. * Values of *p* < 0.05 vs. control, ** *p* < 0.01 vs. control, *** *p* < 0.001 vs. control; ^##^ *p* < 0.01 vs. agomelatine, ^###^ *p* < 0.001 vs. agomelatine. ^†^ *p* < 0.05 vs. HCT-116, ^††^ *p* < 0.01 vs. HCT-116, ^†††^ *p* < 0.001 vs. HCT-116.

**Table 3:** Results of the cosinor analysis of Sirt1-gene expression after agomelatine or melatonin treatment in the HCT-116 and HCT-116-p53-null cell lines.

Cell Line	Treatment	PR ^1^	*p*-Value ^2^	Amplitude (A.U.) ^3^	Acrophase (h) ^4^	MESOR (A.U.)
HCT-116	Control	47.58	<0.0001	0.367 ± 0.071	2.80 ± 0.92	1.37 ± 0.15
	Ago 0.5	47.05	<0.0001	0.456 ± 0.084	22.31 ± 1.41 ***	1.23 ± 0.16
	Mel 1	80.65	<0.0001	0.483 ± 0.042	0.52 ± 0.24 ***^,###^	1.96 ± 0.13
HCT-116 p53 null	Control	34.8	0.0009	0.392 ± 0.093	16.42 ± 1.82 ^†††^	3.48 ± 0.17 ^†††^
	Ago 0.5	35.92	0.0006	0.518 ± 0.114	25.48 ± 1.12 ***	3.91 ± 0.18
	Mel 1	62.29	<0.0001	0.766 ± 0.102 **	4.59 ± 0.34 ***^,###^	4.14 ± 0.17

^1^ PR: percentage of rhythm; ^2^ *p*-value: zero-amplitude test; ^3^ A.U.: arbitrary units; ^4^ h: hours. Comparisons of parameters at the different conditions were performed using a 48-h test period. ** *p* < 0.01 vs. control, *** *p* < 0.001 vs. control; ^###^ *p* < 0.001 vs. agomelatine. ^†††^ *p* < 0.001 vs. HCT-116.

**Table 4:** Results of the cosinor analysis of PEX1-gene expression after agomelatine or melatonin treatment in the HCT-116 and HCT-116-p53-null cell lines.

Cell Line	Treatment	PR ^1^	*p*-Value ^2^	Amplitude (A.U) ^3^	Acrophase (h) ^4^	MESOR (A.U.)
HCT-116	Control	38.04	0.0004	45.7 ± 10.2	3.38 ± 0.56	329 ± 7
	Ago 0.5	74.50	<0.0001	70.4 ± 7.4	3.27 ± 0.31	232 ± 5 ***
	Mel 1	67.01	<0.0001	71.6 ± 8.7	32.54 ± 0.93 ***^,###^	368 ± 6 ***^,###^
HCT-116 p53 null	Control	38.90	0.0003	54 ± 12	0.63 ± 0.79 ^††^	591 ± 8 ^†††^
	Ago 0.5	64.23	<0.0001	81 ± 10	3.83 ± 0.39 ***	447 ± 8 ***
	Mel 1	46.83	<0.0001	65 ± 12	1.71 ± 0.62 ^##^	583 ± 9 ^###^

^1^ PR: percentage of rhythm; ^2^ *p*-value: zero-amplitude test; ^3^ A.U.: arbitrary units; ^4^ h: hours. Comparisons of parameters at the different conditions were performed using a 48-h test period *** *p* < 0.001 vs. control; ^##^ *p* < 0.01 vs. agomelatine, ^###^ *p* < 0.001 vs. agomelatine. ^††^ *p* < 0.01 vs. HCT-116, ^†††^ *p* < 0.001 vs. HCT-116.

## Data Availability

Data is contained within the article and Supplementary Materials.

## Supplementary Materials

The following supporting information can be downloaded at: https://www.mdpi.com/article/10.3390/antiox12040926/s1. Figure S1: Growth inhibition of HCT-116 and HCT-116-p53-null cells after treatment with (a) (c) agomelatine (0–1 mM) and (b) (d) melatonin (0–1 mM) for 24, 48, and 72 h; Figure S2: Raw data of cell-cycle analysis by flow cytometry in HCT-116 cell line after treatment with agomelatine or melatonin; Figure S3: Raw data of cell-cycle analysis by flow cytometry in HCT-116-p53-null cell line after treatment with agomelatine or melatonin; Figure S4: Raw data of apoptosis analysis by flow cytometry in HCT-116 cell line after treatment with agomelatine or melatonin; Figure S5: Raw data of apoptosis analysis by flow cytometry in HCT-116 cell line after treatment with agomelatine or melatonin; Figure S6: Levels of glucose (a), alanine aminotransferase (ALT) (b), amylase (c), urea (d), aspartame aminotransferase (e), and uric acid (f) in sera of nude mice (controls and those treated with agomelatine, melatonin, or 5-FU, as described in Materials and Methods); Table S1: Primers used in the real-time PCR assay; Table S2: Results at 24 h of the cosinor analysis of the Clock genes’ expression after agomelatine or melatonin treatment in the HCT-116 cell line; Table S3: Results at 24 h of the cosinor analysis of the Clock genes’ expression after agomelatine or melatonin treatment in the HCT-116-p53-null cell line; Table S4: Results at 24 h of the cosinor analysis of SIRT1 gene’s expression after agomelatine or melatonin treatment in the HCT-116 and HCT-116-p53-null cell lines; Table S5: Results at 24 h of the cosinor analysis of PRX1 gene expression after agomelatine or melatonin treatment in the HCT-116 and HCT-116-p53-null cell line; Table S6: Median Ct values obtained in the circadian rhythmicity analysis for each gene under all conditions included in the study in HCT-116 cells; Table S7: Median Ct values obtained in the circadian rhythmicity analysis for each gene under all conditions included in the study in HCT-116-p53-null cells.

## Abbreviations

5-FU	5-fluorouracil
Ago	agomelatine
ALT	alanine transaminase
ARE	anti-oxidant-response element
BMAL	brain and muscle ARNT-like
Clock	circadian locomotor cycles kaput
CRC	colorectal cancer
Cry	cryptocrome
CSCs	cancer stem cells
Cul3	cullin 3
Keap1	kelch-like ECH-associated protein 1
Mel	melatonin
MESOR	midline estimating statistic of rhythm
MT	melatonin receptor
NAD	nicotinamide adenine dinucleotide
Nr1d2	nuclear receptor subfamily 1 group D member 2
Nrf2	nuclear factor erythroid 2 related factor-2
Per	period
PR	percentage of rhythm
Prx-1	peroxiredoxin-1
RORα	RAR-related orphan receptor alpha
SIRT1	sirtuin 1
TTFL	transcription–translation feedback loops.
