# Supplementary material for: Agomelatine, a Melatonin-Derived Drug, as a New Strategy for the Treatment of Colorectal Cancer
Source: Antioxidants (Basel). 2023 Apr 13;12(4):926. doi: 10.3390/antiox12040926 (PMC10135458; doi:10.3390/antiox12040926)
Supplement: Supplementary file 1 [file antioxidants-12-00926-s001.zip › antioxidants-2243703-supplementary.pdf]

SUPPLEMENTARY

Table S1: primers used in this study

Gen	Gene Accession Number	Forward	Reverse
Per1	NM_002616.3	ttgatgtgatggcctgtgtggactgtggg	gtgcagtttcctgctgtaggtaaggctgg
Per2	NM_022817.3	ttcttccgttggaaccacccagacatctgc	ccctggtccctttttaagcctcatagtcttgg
Per3	NM_001289862.2	ggggactgcaaaagagaaaacgtccaggcg	aaaaggtcgattttcctaatcattatcaggacgc
Cry1	NM_001413465.1	gtgcgtaatccaaattggtattgtagc	acatgatacttccacaagtttgggtc
Clock	NM_001267843.2	ctatgaggtatgatgtgctgctgggaatgg	aaaatcaaggcactatggggttttttcccctg
Bmal1	NM_001178.6	gcaaatggtcatttcagatgtatgg	ggattcttacaaggaagaataaacgg
Nr1d2	NM_005126.5	acttgaagctattctggaactaacat	catgtcctcatcaattacagtttgtag
Sirt1	NM_012238.5	tagacacgctggaacaggttgc	ctcctcgtacagcttcacagtc
Prx1	NM_002574.4	gggtcttaaaggctgatgaaggcatctcg	agctgggcacacttccccatgtttgtc
UBC	NM_001135592.2	tgggatgcaaatcttcgtgaagaccctgac	accaagtgcagagtggactctttctggatg

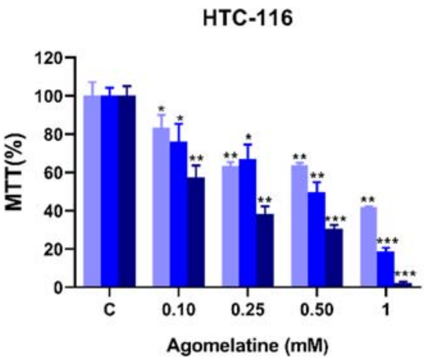

(a)

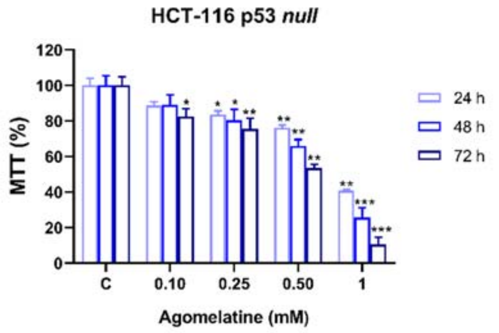

(b)

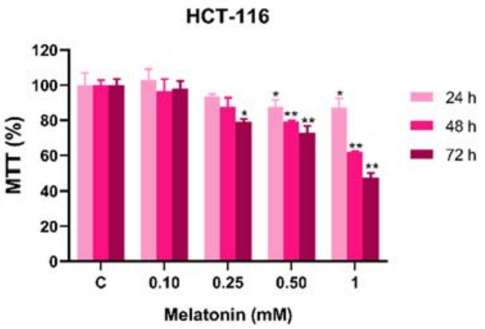

(c)

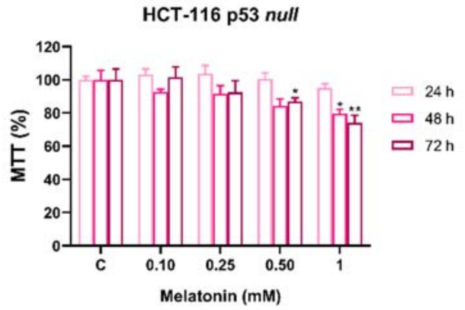

(d)

Figure S1. Growth inhibition of HCT-116 and HCT-116 p53 null cells after treatment with (a) (c) agomelatine (0-1 mM) and (b) (d) melatonin (0-1 mM) for 24, 48 and 72 hours. The results represented the mean ± SD of 3 experiments performed in quadruplicate. *P<0.05 vs C; **P<0.01 vs C; ***P<0.001 vs C.

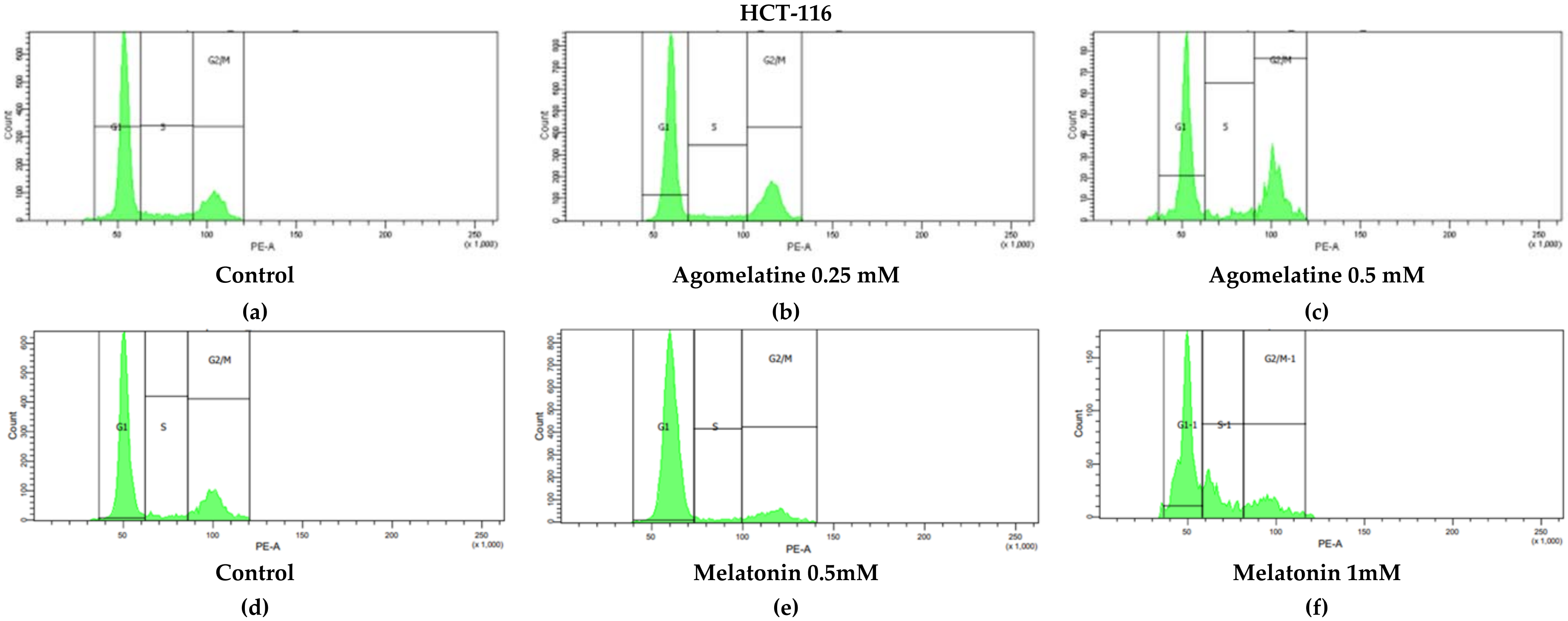

Figure S2. Raw data of cell cycle analysis by flow cytometry in HCT-116 cell line after treatment with agomelatine or melatonin.

HCT-116 p53 null

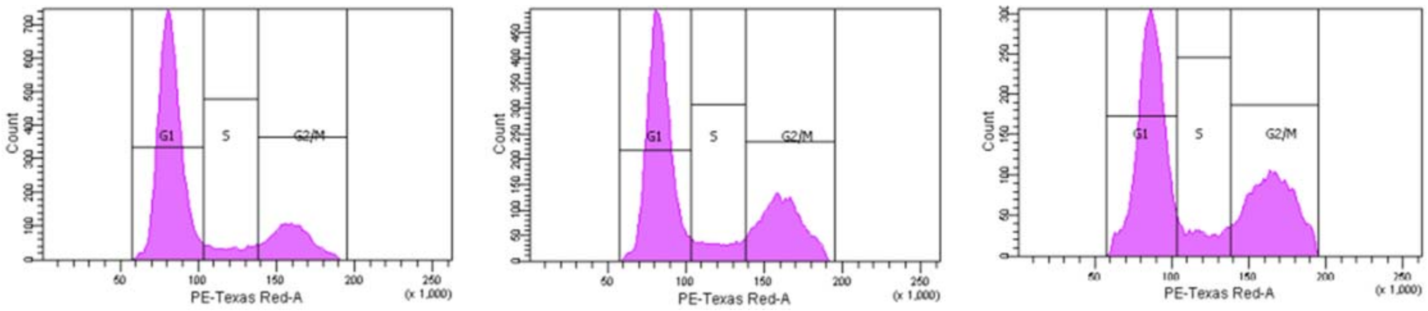

Control

(a)

Agomelatine 0.25 mM

(b)

Agomelatine 0.5 mM

(c)

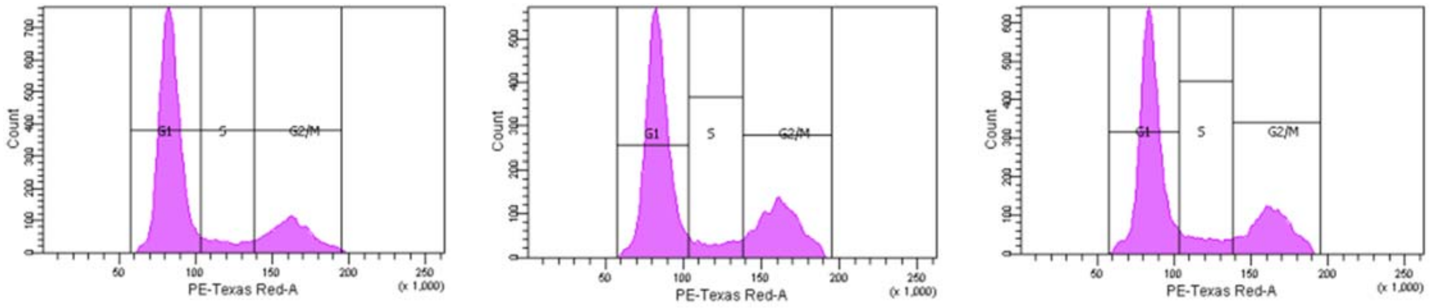

Control

(d)

Melatonin 0.5mM

(e)

Melatonin 1mM

(f)

Figure S3. Raw data of cell cycle analysis by flow cytometry in HCT-116 p53 null cell line after treatment with agomelatine or melatonin.

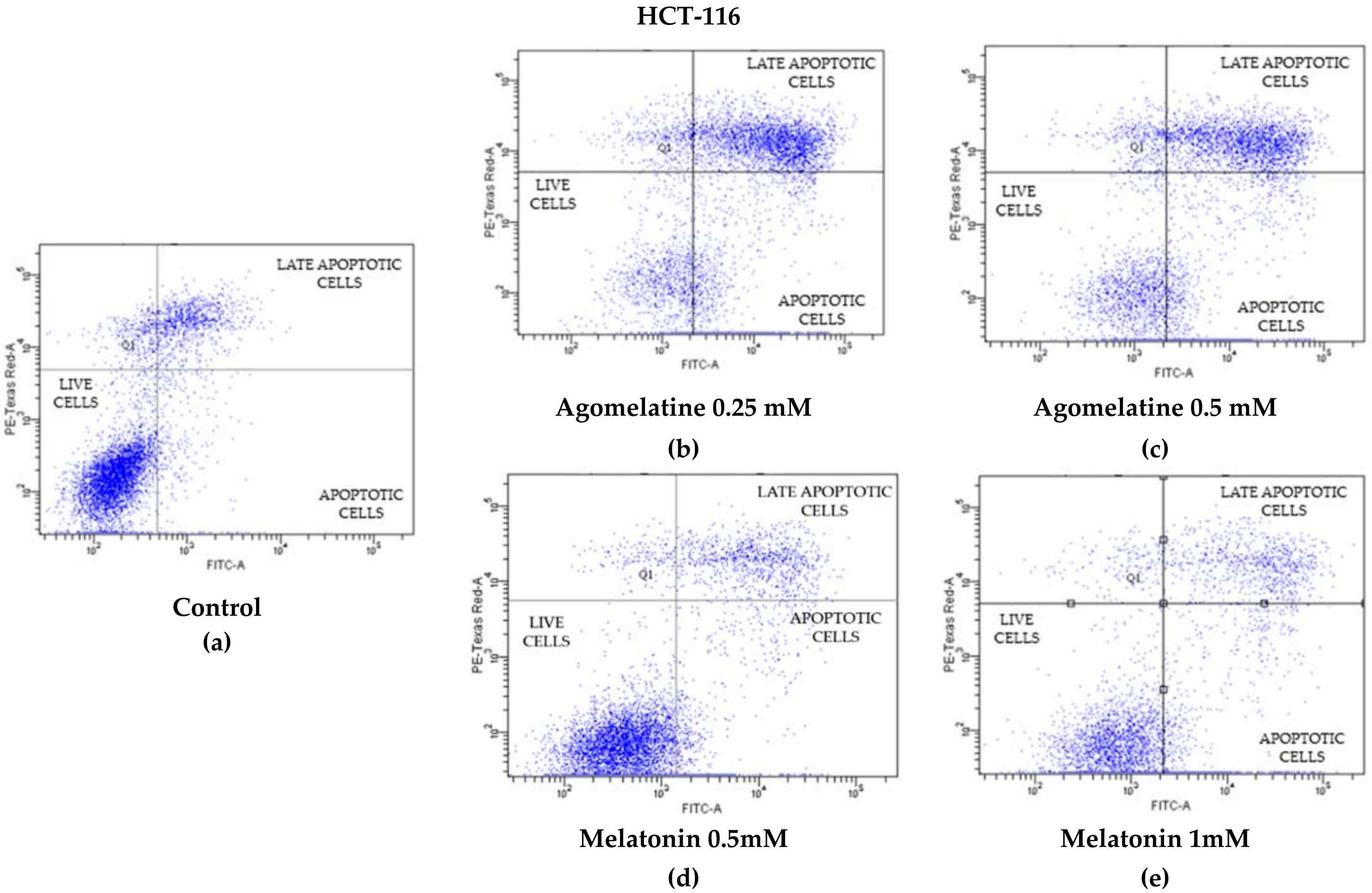

Figure S4. Raw data of apoptosis analysis by flow cytometry in HCT-116 cell line after treatment with agomelatine or melatonin.

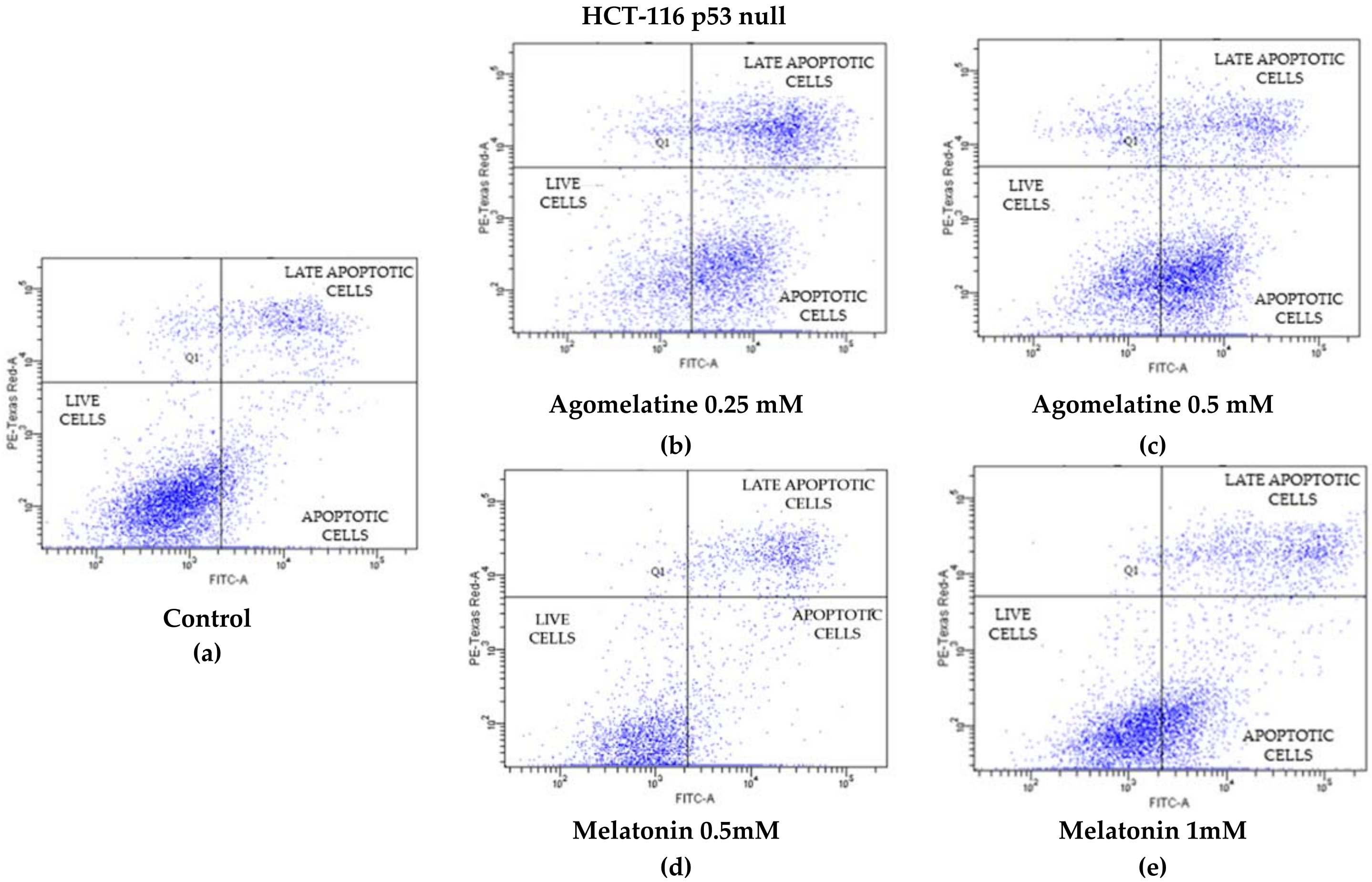

Figure S5. Raw data of apoptosis analysis by flow cytometry in HCT-116 cell line after treatment with agomelatine or melatonin.

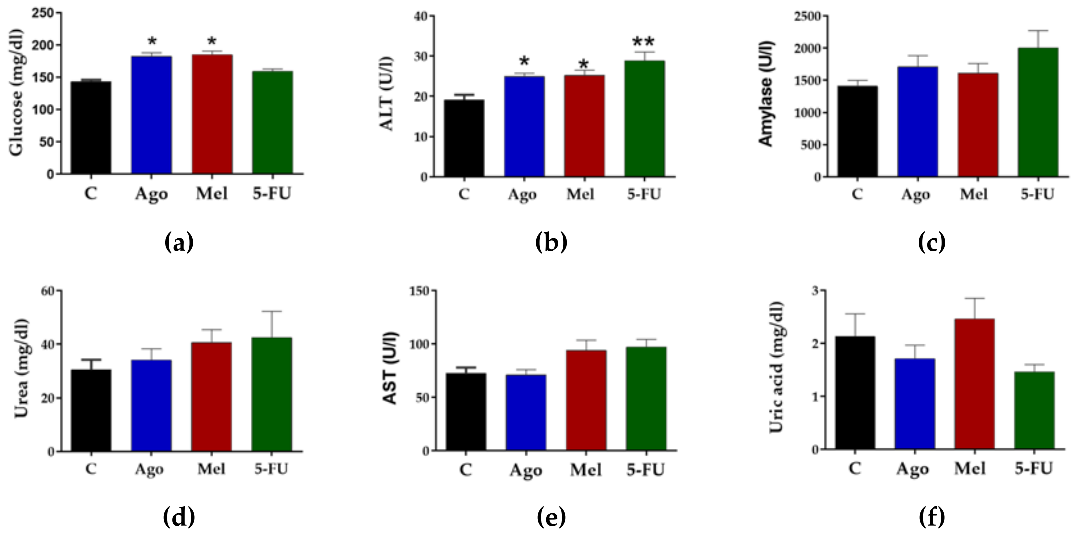

Figure S6. Levels of glucose (a), alanine aminotransferase (ALT) (b), amylase (c), urea (d), aspartame aminotransferase (e) and uric acid (f) in blood, present after the sacrifice of the animals. * P<0.05 C vs. Ago/Mel; **P<0.01 C vs. 5-FU.

Table S2. Results at 24 hours of the cosinor analysis of the clock genes expression after agomelatine or melatonin treatments in the HCT-116 cell line.

Gene	Treatment	PR[1]	P-value[2]	Amplitude (A.U.)[3]	Acrophase (h)[4]	MESOR (A.U.)
**Per1**	Control	81.32	<0.0001	1.63 ± 0.20	3.38 ± 0.95	5.93 ± 0.143
	Ago 0.5	45.07	0.0112	6.06 ± 1.73*	10.24 ± 2.18*	8.49 ± 1.22*
	Mel 1	72.88	0.0001	4.78 ± 0.75*	5.46 ± 0.86	6.84 ± 1.06
**Per2**	Control	73.84	<0.0001	0.087 ± 0.013	1.69 ± 0.59	0.293 ± 0.009
	Ago 0.5	59.78	0.0011	0.155 ± 0.033	8.75 ± 0.81	0.343 ± 0.023***
	Mel 1	35.93	0.0355	0.118 ± 0.041	5.39 ± 1.32	0.305 ± 0.029$^{**,\#}$
**Per3**	Control	76.86	<0.0001	0.73 ± 0.10	6.76 ± 0.54	0.83 ± 0.07
	Ago 0.5	62.12	0.0007	1.09 ± 0.22	7.05 ± 0.77	1.25 ± 0.16
	Mel 1	63.79	0.0005	1.70 ± 0.33$^{**,\#}$	7.07 ± 0.74	1.47 ± 0.23*
**Cry1**	Control	74.52	<0.0001	2.03 ± 0.31	6.34 ± 0.96	3.78 ± 0.22
	Ago 0.5	67.37	0.0002	2.18 ± 0.39	9.78 ± 1.76*	4.15 ± 0.28
	Mel 1	72.36	0.0001	3.19 ± 0.51*	6.80 ± 1.09	4.79 ± 0.36$^{\#}$
**Clock**	Control	8.75	0.5033	0.021 ± 0.018	6.91 ± 5.77	0.265 ± 0.012
	Ago 0.5	63.61	0.0005	0.108 ± 0.021*	3.49 ± 0.68	0.195 ± 0.015*
	Mel 1	28.22	0.0832	0.110 ± 0.046*	7.07 ± 2.91	0.400 ± 0.032$^{***,\#\#\#}$
**Bmal1**	Control	44.46	0.0121	0.0053 ± 0.0029	13.25 ± 3.83	0.020 ± 0.001
	Ago 0.5	18.65	0.2126	0.0059 ± 0.0029	11.61 ± 6.25	0.036 ± 0.002***
	Mel 1	3.00	0.7956	0.0023 ± 0.0034	8.44 ± 12.41	0.023 ± 0.002$^{\#\#\#}$
**Nr1d2**	Control	74.52	<0.0001	2.10 ± 0.43	10.11 ± 2.14	4.21 ± 0.31
	Ago 0.5	67.37	0.0002	1.23 ± 0.16*	3.76 ± 0.47	2.42 ± 0.11***
	Mel 1	72.36	0.0001	1.48 ± 0.27	20.70 ± 3.83$^{***,\#\#}$	2.28 ± 0.19***

[1]PR: Percentage of rhythm; [2] P-value: zero amplitude test; [3]A.U.: arbitrary units; [4]h: hours; * P<0.05 vs control, **P<0.01 vs control, ***P<0.001 vs control; #P<0.05 vs agomelatine, ##P<0.01 vs agomelatine, ###P<0.001 vs agomelatine.

Table S3. Results at 24 hours of the cosinor analysis of the clock genes expression after agomelatine or melatonin treatments in the HCT-116 p53 null cell line.

**Gene**	**Treatment**	**PR**[1]	**P-value**[2]	**Amplitude (A.U.)**[3]	**Acrophase (h)**[4]	**MESOR (A.U.)**
**Per1**	Control	51.93	0.0041	1.77 ± 0.49*	3.49 ± 1.12	4.86 ± 0.33†
	Ago 0.5	39.04	0.0244	5.87 ± 1.89	3.23 ± 0.82	7.46 ± 1.34*
	Mel 1	81.12	<0.0001	2.42 ± 0.31#	4.08 ± 0.52	5.87 ± 0.22
**Per2**	Control	73.63	<0.0001	0.282 ± 0.042†	5.65 ± 0.95†	0.386 ± 0.032†
	Ago 0.5	50.15	0.0054	0.271 ± 0.069	5.96 ± 1.53	0.329 ± 0.050
	Mel 1	60.68	0.0009	0.327 ± 0.064	6.70 ± 1.50	0.365 ± 0.049
**Per3**	Control	81.66	<0.0001	0.181 ± 0.022††	5.42 ± 0.66	0.378 ± 0.015††
	Ago 0.5	15.86	0.2739	0.044 ± 0.027***	5.19 ± 3.09	0.252 ± 0.019***
	Mel 1	83.65	<0.0001	0.181 ± 0.021###	5.76 ± 0.66	0.307 ± 0.015**,#
**Cry1**	Control	90.30	<0.0001	2.70 ± 0.22	5.24 ± 0.46	4.57 ± 0.16†
	Ago 0.5	91.51	<0.0001	2.64 ± 0.21	9.55 ± 0.75***	4.06 ± 0.15*
	Mel 1	95.49	<0.0001	2.69 ± 0.15	6.15 ± 0.35###	4.22 ± 0.11
**Clock**	Control	61.31	0.0008	0.049 ± 0.010	6.42 ± 1.32	0.198 ± 0.007††
	Ago 0.5	39.02	0.0002	0.059 ± 0.011	5.24 ± 0.95	0.207 ± 0.077
	Mel 1	50.92	0.0048	0.053 ± 0.013	6.61 ± 1.67	0.235 ± 0.009
**Bmal1**	Control	32.37	0.0532	0.0036 ± 0.0013	16.53 ± 0.62	0.013 ± 0.001††
	Ago 0.5	4.06	0.7328	0.0012 ± 0.0014	4.97 ± 0.63	0.014 ± 0.001***
	Mel 1	7.02	0.5792	0.0019 ± 0.0018	2.24 ± 2.11	0.014 ± 0.001***
**Nr1d2**	Control	92.54	<0.0001	1.32 ± 0.09	2.34 ± 0.17†	2.51 ± 0.06††
	Ago 0.5	83.42	<0.0001	1.33 ± 0.15	1.85 ± 0.21*	2.64 ± 0.11*
	Mel 1	73.99	<0.0001	1.20 ± 0.18	0.59 ± 0.09***,###	2.79 ± 0.13

[1]PR: Percentage of rhythm; [2] P-value: zero amplitude test; [3]A.U.: arbitrary units; [4]h: hours; * P<0.05 vs control, **P<0.01 vs control, ***P<0.001 vs control; #P<0.05 vs agomelatine, ##P<0.01 vs agomelatine, ###P<0.001 vs agomelatine. †P<0.05 vs HCT-116, †† P<0.01 vs HCT-116.

Table S4. Results at 24 hours of the cosinor analysis of Sirt1 gene expression after agomelatine or melatonin treatments in the HCT-116 and HCT-116 p53 null cell lines.

**Cell line**	**Treatment**	**PR**[1]	**P-value**[2]	**Amplitude (A.U.)**[3]	**Acrophase (h)**[4]	**MESOR (A.U.)**
**HCT-116**	Control	19.34	0.1995	0.256 ± 0.135	4.13 ± 2.01	1.38 ± 0.09
	Ago 0.5	38.31	0.0267	0.170 ± 0.056	21.12 ± 1.25***	1.41 ± 0.04
	Mel 1	81.95	<0.0001	0.488 ± 0.059*,#	0.23 ± 0.03*,###	1.97 ± 0.04***,###
**HCT-116 p53 null**	Control	34.47	0.042	0.255 ± 0.090	18.83 ± 6.71	3.79 ± 0.06†††
	Ago 0.5	95.96	<0.0001	0.908 ± 0.048***	21.61 ± 1.09	3.60 ± 0.34
	Mel 1	93.55	<0.0001	1.050 ± 0.070###	2.93 ± 0.21**,##	4.21 ± 0.05#

[1]PR: Percentage of rhythm; [2] P-value: zero amplitude test; [3]A.U.: arbitrary units; [4]h: hours; * P<0.05 vs control, **P<0.01 vs control, ***P<0.001 vs control; #P<0.05 vs agomelatine, ##P<0.01 vs agomelatine, ###P<0.001 vs agomelatine. ††† P<0.001 vs HCT-116.

Table S5. Results at 24 hours of the cosinor analysis of Prx1 gene expression after agomelatine or melatonin treatments in the HCT-116 and HCT-116 p53 null cell lines.

	Treatment	PR[1]	P-value[2]	Amplitude (A.U.)[3]	Acrophase (h)[4]	MESOR (A.U.)
**HCT-116**	Control	82.62	<0.0001	67.2 ± 8.2	3.35 ± 0.49	299 ± 6
	Ago 0.5	76.24	<0.0001	61.5 ± 8.9	2.56 ± 0.44	235 ± 6***
	Mel 1	68.02	0.0002	60.5 ± 10.7	21.88 ± 0.21***,###	336 ± 8*
**HCT-116 p53 null**	Control	80.94	<0.0001	83 ± 11	0.85 ± 0.11††	464 ± 13†††
	Ago 0.5	67.76	0.0002	99 ± 17	3.77 ± 0.73***	576 ± 7***
	Mel 1	75.83	<0.0001	108 ± 2	1.22 ± 0.68###	586 ± 12###

[1]PR: Percentage of rhythm; [2] P-value: zero amplitude test; [3]A.U.: arbitrary units; [4]h: hours; * P<0.05 vs control, ***P<0.001 vs control; ###P<0.001 vs agomelatine. †† P<0.01 vs HCT-116, ††† P<0.001 vs HCT-116.

Table S6. Median of Ct values obtained in the circadian rhythmicity analysis for each gene at all conditions included in the study in HCT-116 cells.

	**Gene**																										
	**Per1**			**Per2**			**Per3**			**Cry1**			**Clock**			**Bmal1**			**Nr1d2**			**Sirt1**			**Prx1**		
**Time (h)**	**C**	**Mel**	**Ago**	**C**	**Mel**	**Ago**	**C**	**Mel**	**Ago**	**C**	**Mel**	**Ago**	**C**	**Mel**	**Ago**	**C**	**Mel**	**Ago**	**C**	**Mel**	**Ago**	**C**	**Mel**	**Ago**	**C**	**Mel**	**Ago**
**4**	22.56	22.72	21.79	26.44	26.43	26.05	26.10	25.71	25.26	22.80	22.33	22.99	27.46	27.32	27.65	31.48	30.87	31.18	24.50	24.11	23.65	24.46	24.38	25.27	17.08	17.02	17.11
**8**	22.82	22.19	21.68	26.62	27.03	26.20	26.18	25.92	25.33	23.38	22.91	23.40	27.66	27.16	27.99	31.84	30.96	31.09	25.12	24.92	23.91	25.17	25.13	25.08	17.45	17.24	17.71
**12**	23.29	22.11	22.34	27.22	26.82	26.34	26.24	26.45	25.64	23.52	23.10	22.88	28.00	27.13	27.75	31.08	30.73	30.46	25.55	25.05	25.68	25.67	24.85	25.55	17.55	17.60	18.11
**16**	22.68	22.54	22.49	28.07	28.54	27.33	27.83	27.49	27.19	24.35	24.08	23.47	27.62	27.47	28.09	31.41	31.07	30.46	24.47	24.04	24.79	25.07	24.37	24.91	17.23	16.92	17.58
**20**	22.53	22.68	22.97	27.72	28.60	28.20	27.91	28.40	26.85	24.59	24.51	24.53	27.90	27.86	27.91	31.68	31.75	31.25	23.98	23.92	24.44	25.21	24.47	25.02	16.82	16.87	17.46
**24**	22.23	21.91	23.25	26.86	27.36	28.47	27.04	28.69	26.98	24.15	24.33	25.12	28.07	27.75	28.53	32.41	31.67	31.38	24.11	23.70	23.91	25.66	24.68	24.96	17.32	16.98	17.51
**28**	23.07	21.96	22.63	27.84	27.76	28.36	27.36	28.57	27.67	24.02	24.07	24.77	29.47	29.25	29.29	33.84	33.75	32.46	24.69	24.54	23.88	25.13	24.79	24.84	17.12	16.73	17.78
**32**	22.78	22.36	22.27	28.05	28.64	26.87	27.86	27.01	25.66	23.74	23.80	23.72	29.20	29.16	29.59	33.18	33.47	32.38	25.50	25.36	24.10	25.10	24.85	25.20	16.85	16.75	17.95
**36**	22.60	22.85	21.91	28.25	27.62	26.87	26.45	27.01	25.50	23.89	23.24	23.10	29.05	28.69	28.84	32.54	31.70	31.96	25.09	24.39	24.12	24.61	24.37	25.37	16.82	16.56	18.03
**40**	22.16	22.92	22.78	28.17	27.96	26.37	27.07	28.02	27.21	24.20	23.82	23.33	28.70	28.80	28.63	32.12	32.15	31.64	24.43	24.26	23.94	25.08	24.30	25.04	16.92	16.80	17.07
**44**	22.63	22.55	20.74	26.89	28.18	26.53	26.89	29.69	28.66	23.42	24.19	24.54	29.75	29.65	29.65	32.86	32.77	31.16	23.60	23.54	23.75	25.70	25.09	25.84	17.24	17.10	17.38
**48**	22.86	22.39	20.85	26.78	27.33	29.16	26.71	29.41	28.26	22.97	23.46	24.77	30.10	28.99	30.01	33.59	32.69	31.44	24.25	23.63	23.43	25.66	25.01	26.43	17.26	17.11	18.25

Table S7. Median of Ct values obtained in the circadian rhythmicity analysis for each gene at all conditions included in the study in HCT-116 p53 null cells.

	**Gene**																										
	**Per1**			**Per2**			**Per3**			**Cry1**			**Clock**			**Bmal1**			**Nr1d2**			**Sirt1**			**Prx1**		
**Time (h)**	**C**	**Mel**	**Ago**	**C**	**Mel**	**Ago**	**C**	**Mel**	**Ago**	**C**	**Mel**	**Ago**	**C**	**Mel**	**Ago**	**C**	**Mel**	**Ago**	**C**	**Mel**	**Ago**	**C**	**Mel**	**Ago**	**C**	**Mel**	**Ago**
**4**	22.75	22.72	21.91	26.48	26.27	26.67	26.34	26.59	26.95	22.65	22.88	23.30	28.16	27.67	27.90	32.14	31.50	31.61	24.18	24.02	23.81	23.35	23.03	23.86	16.17	16.02	16.18
**8**	23.31	23.05	22.18	26.74	26.55	27.51	26.84	26.75	28.08	23.06	22.94	23.05	28.03	27.70	28.13	32.63	31.77	32.02	24.74	24.11	24.21	23.97	23.55	24.27	16.47	16.54	16.80
**12**	23.37	22.91	23.04	27.84	27.93	27.35	27.16	27.73	27.67	23.65	23.69	23.12	28.36	28.29	28.42	32.12	31.74	31.09	25.69	25.44	25.56	23.77	24.13	24.09	16.71	16.69	16.94
**16**	23.40	23.34	23.11	28.90	29.13	28.80	28.00	28.42	27.94	24.45	24.59	23.60	28.44	28.14	28.49	31.37	31.86	30.69	24.66	24.76	24.74	23.67	23.66	23.88	16.44	16.43	16.85
**20**	22.90	22.97	23.80	27.89	28.61	29.05	27.74	27.95	27.79	24.33	24.65	24.97	28.60	28.35	28.16	32.23	31.74	31.33	23.94	23.84	24.44	23.63	23.32	23.48	16.27	16.21	16.65
**24**	22.33	22.24	22.95	27.13	27.78	28.87	27.18	27.77	28.00	23.61	23.84	25.37	28.27	28.41	27.75	32.25	32.41	30.94	23.73	23.74	23.69	23.87	23.47	23.59	16.29	16.27	16.54
**28**	22.64	23.05	22.16	27.27	27.89	28.48	26.45	26.54	27.48	23.23	23.83	24.89	28.98	29.09	28.90	31.78	31.74	31.40	23.47	23.47	23.38	24.03	24.08	23.63	16.26	16.41	16.96
**32**	22.80	22.75	21.85	27.82	27.93	27.24	27.00	27.50	26.89	23.33	23.56	24.03	28.95	28.62	28.76	31.35	30.97	30.97	23.64	24.01	22.71	24.10	23.58	23.68	16.52	16.33	16.97
**36**	23.01	23.57	22.17	28.31	29.29	27.19	27.64	27.74	26.64	24.68	24.19	23.58	29.44	28.80	28.28	30.96	30.76	30.66	23.72	23.72	22.70	23.71	23.76	23.58	16.33	16.56	18.03
**40**	22.59	22.63	22.53	28.35	29.07	27.44	27.62	28.03	26.99	24.66	24.64	23.62	29.15	29.02	28.07	30.85	30.64	30.41	23.49	23.40	22.95	23.99	23.69	23.57	16.21	16.80	17.07
**44**	22.14	22.60	24.29	26.90	27.14	27.62	26.35	26.35	25.90	23.49	23.86	24.00	28.98	28.21	28.12	31.43	30.72	30.43	23.01	23.04	22.64	24.29	23.75	24.00	16.47	17.10	17.38
**48**	22.04	22.64	23.07	27.27	26.62	28.17	27.74	27.41	26.53	23.30	23.19	24.56	29.58	28.50	28.75	31.61	31.05	30.83	23.92	23.19	22.75	24.09	23.42	23.63	16.14	17.11	18.25
